# A Novel *Salmonella* Periplasmic Protein Controlling Cell Wall Homeostasis and Virulence

**DOI:** 10.3389/fmicb.2021.633701

**Published:** 2021-02-19

**Authors:** Juan J. Cestero, Sónia Castanheira, M. Graciela Pucciarelli, Francisco García-del Portillo

**Affiliations:** ^1^Laboratory of Intracellular Bacterial Pathogens, National Centre for Biotechnology (CNB)-CSIC, Madrid, Spain; ^2^Department of Molecular Biology, Autonomous University of Madrid, Madrid, Spain; ^3^Center for Molecular Biology “Severo Ochoa” (CBMSO)-CSIC, Madrid, Spain

**Keywords:** *Salmonella*, peptidoglycan, periplasm, MltD, regulation

## Abstract

Horizontal gene transfer has shaped the evolution of *Salmonella enterica* as pathogen. Some functions acquired by this mechanism include enzymes involved in peptidoglycan (PG) synthesis and remodeling. Here, we report a novel serovar Typhimurium protein that is absent in non-pathogenic bacteria and bears a LprI functional domain, first reported in a *Mycobacterium tuberculosis* lipoprotein conferring lysozyme resistance. Based on the presence of such domain, we hypothesized a role of this *S.* Typhimurium protein in PG metabolism. This protein, which we named ScwA for *Salmonella* cell wall-related regulator-A, controls positively the levels of the murein lytic transglycosylase MltD. In addition, the levels of other enzymes that cleave bonds in the PG lattice were affected in a mutant lacking ScwA, including a soluble lytic tranglycosylase (Slt), the amidase AmiC, and a few endo- and carboxypeptidases (NlpC, PBP4, and AmpH). The *scwA* gene has lower G+C content than the genomic average (43.1 vs. 52.2%), supporting acquisition by horizontal transfer. ScwA is located in the periplasm, stabilized by two disulfide bridges, produced preferentially in stationary phase and down-regulated following entry of the pathogen into eukaryotic cells. ScwA deficiency, however, results in a hypervirulent phenotype in the murine typhoid model. Based on these findings, we conclude that ScwA may be exploited by *S.* Typhimurium to ensure cell envelope homeostasis along the infection and to prevent host overt damage. This role could be accomplished by controlling the production or stability of a reduced number of peptidoglycan hydrolases whose activities result in the release of PG fragments.

## Introduction

*Salmonella enterica* is one of the most successful bacterial pathogens known causing with high morbidity and mortality food-borne diseases in humans and livestock (Ao et al., 2015; Haselbeck et al., 2017; Gibani et al., 2018; G. B. D. Non-Typhoidal Salmonella Invasive Disease Collaborators, 2019). Despite the large diversity of subspecies and serovars known in *S. enterica*, with many of them exhibiting distinct host range (Baumler and Fang, 2013), they share some traits in their pathogenicity strategies. Thus, *S. enterica* evolved as a pathogen following acquisition of genomic islands that encode specialized type III secretion systems (T3SS). These virulence-related T3SS allow bacteria to invade and survive inside phagocytic and non-phagocytic cells (Galan, 2001; Galan et al., 2014) by mechanisms involving translocation of effector proteins into the infected host cell that subvert vesicular trafficking and cytoskeletal dynamics (de Souza Santos and Orth, 2015; Personnic et al., 2016; Jennings et al., 2017). The genes encoding structural components of these T3SS apparatuses and effector proteins are present only in pathogenic bacteria and display features that reveal acquisition by horizontal gene transfer, being the more prominent a different G+C% than the genomic average (Gyles and Boerlin, 2014; Ilyas et al., 2017).

Besides effector proteins exploited by *S. enterica* to build a specialized phagosome and to adapt to the intracellular lifestyle, this pathogen modifies the cell envelope to withstand varied host cell defenses, including antimicrobial peptides. Some of these changes involve alterations in the lipid-A portion of the lipopolysaccharide (LPS), modifications that are orchestrated by the functionally interconnected regulatory systems PhoP-PhoQ and PmrA-PmrB (Chen and Groisman, 2013; Dalebroux and Miller, 2014), both required for virulence. Recent evidence also supports the existence of additional modifications in the cell envelope of intracellular *S. enterica*, specifically in the peptidoglycan (PG). Once it is inside eukaryotic cells, *S. enterica* serovar Typhimurium (*S*. Typhimurium) increases the production of EcgA, an D,L-endopeptidase that cleaves the D-glutamic acid-*meso*-diaminopimelic acid (D-Glu-*m*Dap) bond in stem peptides of the PG and that is required for virulence in the mouse typhoid model (Rico-Perez et al., 2016). An augmented cleavage of the D-Glu-*m*Dap bond in the PG of intra-phagosomal bacteria was postulated to attenuate stimulation of intracellular host defense receptors like the nucleotide oligomerization domain (NOD)-family NOD1 receptor (Caruso et al., 2014; Philpott et al., 2014; Rico-Pérez et al., 2016). This evasion mechanism is consistent with the diminished nuclear translocation of the NF-kB p65 subunit and release of proinflammatory cytokines observed in cells persistently infected with *S.* Typhimurium (Ramos-Marques et al., 2017). The structural changes in the PG responding to intracellular host cues are, however, rarely studied in host tissues, with most descriptions reported in pathogens grown in laboratory media (Pucciarelli and Garcia-del Portillo, 2018; Garcia-Del Portillo, 2020). Common modifications found in the PG of bacterial pathogens include O-acetylation in sugars composing the glycan chains (Moynihan et al., 2014; Sychantha et al., 2018; Brott and Clarke, 2019), amidation of amino acid residues in the stem peptide (Munch and Sahl, 2015) or, incorporation of non-canonical D-amino acids (Alvarez et al., 2014). These modifications are known in pathogens like *Helicobacter pylori* (Sycuro et al., 2012; Wang et al., 2012), *Neisseria* sp. (Chan et al., 2012), *Mycobacterium tuberculosis* (Hansen et al., 2014), and *Listeria monocytogenes* (Boneca et al., 2007; Aubry et al., 2011), among others. In most cases, comparative genomics has been a powerful tool to identify enzymes responsible for PG modifications that are absent in non-pathogenic bacteria (Boneca et al., 2007; Aubry et al., 2011).

In addition to EcgA, the *S.* Typhimurium genome encodes penicillin-binding proteins (PBPs) that are up-regulated following the entry into eukaryotic cells. These PBPs, named PBP2_SAL_ and PBP3_SAL_, are absent in its closest phylogenetic bacterium, *Escherichia coli*. Interestingly, PBP2_SAL_ and PBP3_SAL_ contribute to growth and division of *S.* Typhimurium inside host cells, are produced and functional in acidic pH conditions, and replace PBP2 and PBP3 *in vivo* in bacteria colonizing mouse target organs (Castanheira et al., 2017, 2018, 2020). Although the substrate(s) recognized by these two pathogen-specific PBPs remain unknown, their production in infection conditions indicate that they probably contributed to the evolution of *S. enterica* as an intracellular pathogen. PBP3_SAL_ has also been recently related to the increased relapse rate reported in salmonellosis patients treated with third-generation cephalosporins (Castanheira et al., 2020).

In this study, we report a *S.* Typhimurium gene absent in most *E. coli* isolates that encodes a periplasmic protein important to maintain the levels of the murein transglycosylase MltD and, to a lesser extent, of other enzymes that hydrolyze different bonds in the glycan chains or stem peptides of the PG lattice. This new protein, which we termed ScwA for *Salmonella* cell wall-related regulator-A, is also required for fine-tuning the damage inflicted to the host during the infection process.

## Materials and Methods

### Bacterial Strains and Plasmids

The *S.* Typhimurium and *E. coli* strains and plasmids used in this study are listed in Supplementary Table 1. Bacteria were grown at 37°C in Luria-Bertani (LB) broth, composed of 1% (w/v) casein peptone, 0.5% (w/v) yeast extract and 0.5% (w/v) sodium chloride or, in phosphate-carbon-nitrogen (PCN)-limited minimal medium buffered with 80 mM MES [2-(N-morpholino) ethanesulfonic acid]. The composition of PCN medium is: 4 mM Tricine [N-Tris(hydroxymethyl) methyl-glycine], 0.1 mM FeCl_3_, 376 μM K_2_SO_4_, 50 mM NaCl, 15 mM NH_4_Cl, 1 mM MgSO_4_, 1 μM CaCl_2_, 0.4% (w/v) glucose, 0.4 mM inorganic phosphate (P_i_), and micronutrients (Deiwick and Hensel, 1999). The 80 mM MES solution was adjusted to the desired pH value with NaOH. When required, ampicillin (100 μg/mL), kanamycin (30 μg/mL) and chloramphenicol (10 μg/mL), were added to the media.

### DNA Manipulation Techniques

Gene inactivation and 3×FLAG epitope tagging were carried out following one-step protocols, as described (Datsenko and Wanner, 2000; Uzzau et al., 2001). For the RT-PCR assays, RNA from exponential phase cultures was extracted with 5% phenol / 95% ethanol (v/v), as described (McDowall et al., 1994) and treated with RNeasy Mini Kit (Qiagen). The resulting RNA was treated with DNase (2 U/μL) and 1 μg of RNA was transcribed to cDNA using High-Capacity cDNA Reverse Transcription Kit (Applied Biosystems) These assays were performed independently with RNA isolated from two biological replicates. Oligonucleotides used for the RT-PCR assays and the rest of genetic procedures are listed in Supplementary Table 2. The *scwA* gene and its derivatives with point mutations were cloned in the expression vector pUHE21-2*lacI*^*q*^ (Soncini et al., 1995) using *E. coli* DH5α as host. *scwA* was amplified with primers ForBamHIScwAYbaP / RevHindIIIScwA (Supplementary Table 2), digested with BamHI and HindIII and ligated into pUHE21-2*lacI^*q*^.* The different *scwA* variants bearing point mutations were generated using primers ForBamHIScwAYbaP / ScwACys30-SerRv and ScwACys30-SerFw / RevHindIIIScwA for C30S variant and ForBamHIScwAYbaP / ScwACys100-SerRv and ScwACys100-SerFw / RevHindIIIScwA for the C100S variant (Supplementary Table 2). For each variant, two DNA fragments were obtained using the two pair of primers and then combined by annealing and extension. The resulting DNA fragment was amplified by PCR, digested with BamHI and HindIII and ligated into pUHE21-2*lacI*^*q*^. All constructs were sequenced to confirm the absence of undesired mutations.

### Bacterial Subcellular Fractionation

Bacteria (ca. 3 × 10^9^) grown to stationary phase were spun down by centrifugation (14,100 × *g*, 5 min, 4°C) and resuspended in 1 mL of phosphate saline buffer (PBS). Cytosol and inner/outer membrane fractions were prepared as described (Pucciarelli et al., 2002). To prepare fractions enriched in periplasmic proteins, bacteria were spun down (14,100 × g, 10 min, 4°C) and the pellet stored for 30 min at −80°C. After this, 2 mL of pre-warmed PBS were added to each pellet and vigorously mixed. Supernatant was collected after centrifugation (14,100 × *g*, 5 min, 4°C). This procedure was repeated once more, obtaining 4 mL of supernatant that were centrifuged (3,200 × *g*, 15 min, 12°C) and concentrated to a final volume of 250 μL using 15 mL-Amicon 10 K tubes (Merck).

### Alkylation Assay to Infer the Presence of Disulfide Bonds

A 2 mL culture of bacteria producing epitope-tagged ScwA-3×FLAG was centrifuged (14,100 × *g*, 5 min, 4°C) and bacteria resuspended in 2 mL of alkylation buffer (150 mM Tris-HCl pH 7.5, 2% SDS). Samples were sonicated and then centrifuged twice (4,000 × *g*, 5 min, 4°C) to discard unbroken cells. Supernatant was centrifuged at high speed (29,400 × *g*, 20 min, 4°C) obtaining a new supernatant and a pellet which was resuspended in equal volume of alkylation buffer. Both supernatant and pellet were divided in four aliquots of 450 μL each. Then, 50 μL of 100 mM dithiothreitol (DTT) were added to two samples of each group and alkylation buffer to the rest of samples. After 1 h at 37°C, 10% (w/v) trichloroacetic acid (TCA) was added to precipitate proteins by further incubation for 1 h on ice. Precipitated proteins were centrifuged (29,400 × *g*, 15 min, 4°C), washed with 1 mL of acetone and finally dried. Within each group, two samples (+DTT, −DTT) were resuspended in 25 μL alkylation buffer, whereas the remaining samples were resuspended in 25 μL of alkylation buffer containing 15 mM [4-acetamido-4’-maleimidylstilbene-2,2’-disulfonic acid] (AMS). Samples were incubated 1 h at room temperature. Laemmli buffer without β-mercaptoethanol (1.3% SDS, 10% glycerol, 50 mM Tris–HCl pH 6.8, 0.02% bromophenol blue) was finally added. The inner membrane protein IgaA was used as control of protein with disulfide bonds (Pucciarelli et al., 2017). The assays were performed in samples prepared from three independent biological replicates.

### Lysozyme Susceptibility Assay

Overnight cultures of isogenic wild type and Δ*scwA* bacteria were diluted to an initial optical density (OD_600_) of 0.02 in 5 mL of LB broth. At exponential phase (OD_600_ ∼0.2–0.3) and stationary phase (OD_600_ ∼2.5), cultures were centrifuged and the pellet washed with 10 mM phosphate buffered saline (PBS) pH 7.2 and diluted to 5 × 10^5^ CFU/mL. 500 μL of each sample were mixed with 4.95 mL of 10 mM PBS pH 7.2 and divided in four aliquots of 1 mL for the different treatments: control (only buffer), 100 μg/mL lysozyme, 0.1 mM EDTA, 100 μg/mL lysozyme + 0.1 mM EDTA. After an incubation of 1 h at 30°C, 100 μL of 10-fold serial dilutions were plated in LB for colony counting and determination of survival rates. These assays were repeated in a total of four biological replicates.

### Eukaryotic Cell Lines and Bacterial Infection Assays

Fibroblasts NRK-49F (ATCC CRL-1570) and HeLa epithelial cells (ATCC CCL-2) were propagated in Dulbecco’s modified Eagle’s medium (DMEM) containing 10% (v/v) fetal bovine serum (FBS) at 37°C in a 5% CO_2_ atmosphere as described previously (Nunez-Hernandez et al., 2013). Briefly, eukaryotic cells were infected at a MOI (multiplicity of infection) of 10:1 with wild-type or Δ*scwA* isogenic strains, previously grown overnight at 37°C in LB without shaking. At 2, 8, and 24 h post-infection, the infected cultures were lysed in PBS-1% Triton X-100 and the extracts plate on LB-plates for colony counting and determination of viable intracellular bacteria. For large-scale experiments needed to monitor protein production by intracellular bacteria in persistent infections, NRK-49F fibroblasts were grown in 500 cm^2^ plates and infected at a MOI of 10:1 with strain MD5250 (*scwA*::3×FLAG *mltD*::3×FLAG-*kan*) (Supplementary Table 1). At 2, 4, and 8 hpi, infected cells were lysed and processed for western analysis as described (Nunez-Hernandez et al., 2013).

### Determination of Peptidoglycan Enzyme Production

To assess the levels of peptidoglycan (PG) enzymes produced in isogenic wild type and Δ*scwA* strains, 3×FLAG-tagged alleles of the PG enzymes of interest were introduced in these two genetic backgrounds by P22 HT105/1 *int201* phage transduction (Schmieger, 1972). Strains bearing these alleles were diluted to an initial OD_600_ ∼0.02 in LB and grown until stationary phase (OD_600_ ∼2.5). 1×10^9^ bacteria were collected by centrifugation (15,000 × *g*, 10 min, 4°C), washed, resuspended in 75 μL of PBS pH 7.4. Laemmli buffer (4×) was finally added. To determine levels of PG enzymes in strains overproducing wild type ScwA or variants lacking disulfide bridges, overnight cultures of tagged strains containing the respective pUHE21::*scwA* plasmids were diluted to an initial OD_600_ ∼0.02. In exponential phase (OD_600_ ∼0.2–0.3), 1 mM IPTG was added and incubation prolonged for 40 min. As in the wild type and Δ*scwA* bacterial cultures, whole cell lysates were obtained from 1 × 10^9^ cells. These assays were repeated in a minimum of three independent biological replicates.

### Antibodies and Western-Blot Assays

Primary antibodies and their working dilutions used for western blotting included: mouse monoclonal anti-FLAG epitope (clone M2, Sigma) 1:5,000; rabbit polyclonal anti-IgaA (Cano et al., 2002) 1:10,000; rabbit polyclonal anti-OmpA (gift from H. Schwarz, University of Tübingen, Germany) 1:50,000; mouse monoclonal anti-DnaK (Enzo) 1:10,000; mouse monoclonal anti-β-lactamase (gift from L.A. Fernández, CNB-CSIC, Madrid, Spain) 1:2,000; rabbit polyclonal anti-PBP3 (Castanheira et al., 2017) 1:1,000; and, rabbit polyclonal anti-PBP2 (Castanheira et al., 2020) 1:1,000. Goat polyclonal anti-mouse or anti-rabbit IgG conjugated to horseradish peroxidase (Bio-Rad) were used as secondary antibodies at a 1:10,000 dilution. SDS-PAGE and western blotting were performed as described (Nunez-Hernandez et al., 2014).

### Virulence Assays in BALB/c Mice

Intraperitoneal challenge of 8-weeks-old female BALB/c mice was performed based on competition experiments, as described (Dominguez-Bernal et al., 2004). The input mixture was 5 × 10^5^ CFU with the output determined at 48 hpi. Wild type and mutant strains bearing a Km^R^ allele were differentiated plating on LB and LB-kanamycin plates.

Animal experiments were performed in accordance with the guidelines of the European Commission for the handling of laboratory animals (directive 2010/63/EU) and approved by the Environment Council (Consejería de Medio Ambiente) of the Regional Government of Madrid, under license PROEX 110/19.

### Prediction of ScwA Secondary Structure and Protein Folding

Secondary structure of ScwA was predicted using the PSIPRED server^1^. Protein fold prediction and 3-D modelling were obtained in the Phyre-2 server^2^.

### Statistical Analysis

Data were analyzed using GraphPad Prism, version 8.0, software (GraphPad Inc., San Diego, CA, United States). *t*-test, One-way and Two-way ANOVA, this latter followed by Tukey’s post-tests for multiple comparisons, were used for data analysis. Significance was established at *P* values ≤ 0.05. In all cases, a minimum of three independent (biological) replicates were analyzed.

## Results

### Identification of SL1344_0490 (ScwA), a Novel *Salmonella* Protein Related to Peptidoglycan (PG) Metabolism

**FIGURE 1:**
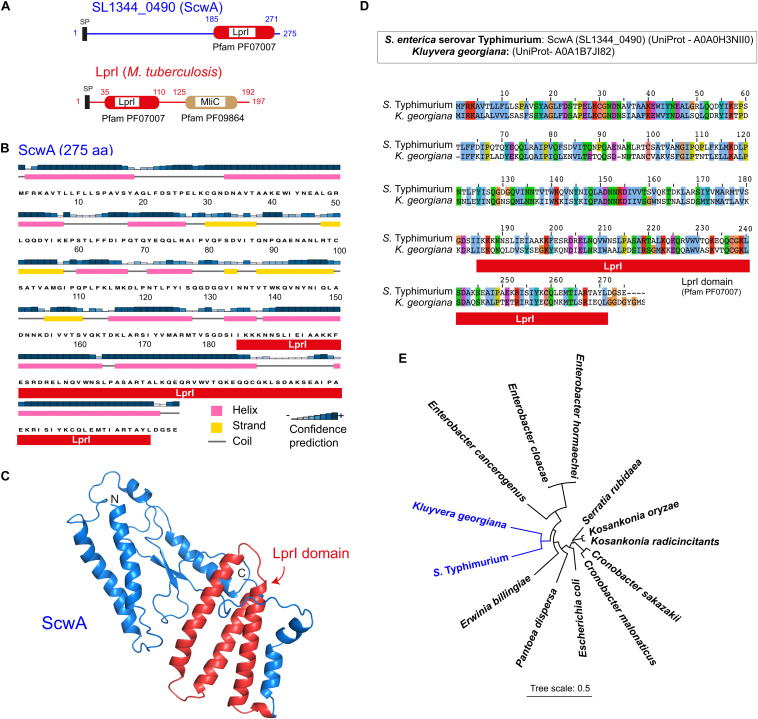
The *S.* Typhimurium protein SL1344_0490 (ScwA) bears a LprI domain and has orthologs restricted to a few enterobacterial genera. **(A)** Domain architecture predicted by SMART (http://smart.embl-heidelberg.de/) in the 275 amino acid protein SL1344_0490 (UniProt entry A0A0H3NII0). Indicated is the LprI (lysozyme inhibitor) domain (Pfam PF07007), encompassing residues 185 to 271. The *M. tuberculosis* lipoprotein LprI, in which the LprI domain was first reported, is shown for comparison. *M. tuberculosis* LprI, unlike SL1344_0490 (ScwA), bears a second domain named MliC for membrane-bound lysozyme inhibitor (Pfam PF09864); SP, signal peptide. **(B)** ScwA secondary structure predicted by the PSIPRED software (http://bioinf.cs.ucl.ac.uk/psipred/), showing helix, strand, and coil regions. **(C)** ScwA folding predicted by Phyre-2 (http://www.sbg.bio.ic.ac.uk/~phyre2). Indicated in red are the three alpha-helices that conform the LprI domain. **(D)** Alignment of ScwA to its closest ortholog, a protein of unknown function from *Kluyvera georgiana*, showing 50% identity. **(E)** Phylogeny tree inferred from the sequences of *S.* Typhimurium ScwA protein and its closest orthologs: *K. georgiana* (A0A1B7JI82); *Erwinia billingae* (WP106382170.1); *Pantoea dispersa* (WP125310743.1); *Escherichia coli* (WP141220346.1); *Cronobacter malonaticus* (WP105624211.1); *Cronobacter sakazakii* (WP080321002.1); *Kosakonia radicincitans* (WP043952829.1); *Kosakonia oryzae* (WP064564758.1); *Serratia rubidaea* (WP0543207953.1) *Enterobacter hormaecheii* (WP148387290.1); *Ent. cloacae* (WP063151490.1); *Ent. cancerogenus* (WP 153688383.1). The tree was generated using iTOL (https://itol.embl.de/).

To identify new pathogen-specific proteins related to PG metabolism, we mined the genome of *S. enterica* serovar Typhimurium (*S.* Typhimurium) reference strain SL1344 for genes fulfilling two criteria: first, to be absent in non-pathogenic *E. coli* and, second, to bear functional domains reported in proteins linked to PG metabolism. The gene *SL1344_0490*, encoding a putative protein of 275 amino acids of unknown function, fulfilled both criteria. The SMART tool^3^ predicted in SL1344_0490 (UniProt A0A0H3NII0) a LprI domain spanning from residues 185 to 271 with an *E*-value of 3.6×10^–11^ (Figure 1A). The LprI domain (Pfam PF07007) was first reported in a lipoprotein -also named LprI- present exclusively in pathogenic mycobacteria and proposed to confer lysozyme resistance (Sethi et al., 2016). LprI of *Mycobacterium tuberculosis* bears a second domain, MliC, which attains for membrane-bound lysozyme inhibitor (Pfam PF09864). This MliC domain is, however, missing in SL1344_0490 (Figure 1A). Secondary structure and protein folding predictions showed that SL1344_0490, which we renamed ScwA for *Salmonella* cell wall-related regulator-A (see below), is a protein with a high content of alpha-helices and with the LprI domain exposed on the surface of the protein (Figures 1B,C). Considering a potential link of ScwA to PG metabolism based on the presence of this LprI domain, we pursued its further study.

BLASTP search showed that ScwA is conserved in the *S. enterica* species and, in addition, present in eight clinical and host-associated isolates of the non-pathogenic species *S. bongori* (Supplementary Figure 1). Outside the *Salmonella* genus, the highest homology to ScwA was found in a 278 amino acid protein of unknown function from *Kluyvera georgiana* annotated as “DUF1311 domain-containing protein” (UniProt A0A1B7JI82). This highest homology accounted, however, only for a 50% identity uniformly distributed along the protein (Figure 1D). Orthologs with lower identities but with homologies equally spanning along the full sequence were identified in other enteric bacteria, including *Enterobacter hormaechei* (39–40% identity), *Kosakonia oryzae* (39%), *Kosakonia radicincitans* (39%), *Enterobacter cloacae* (38–39%), *Serratia rubidaea* (38%), *Enterobacter cancerogenus* (37%), *Cronobacter sakazakii* (36–37%), *C. malonaticus* (37%), *Erwinia billingiae* (38%), and *Pantoea dispersa* (35%). Interestingly, ScwA orthologs were also found in 22 *E. coli* strains that, as in *S. bongori*, were mostly associated to disease, isolated from food, feces of infected animals or urinary infections in humans (Supplementary Figure 2). The identity at the amino acid level in these ScwA orthologs of such few *E. coli* isolates was in all cases of 37–38%, also spanning along the entire protein sequence (>95% coverage) (Supplementary Figure 2). Orthologs found in these enteric bacteria have a similar size compared to *S.* Typhimurium ScwA, in the range of 275–278 amino acids. Consistently with the BLASTP results, the phylogenetic analysis of these putative orthologs confirmed that ScwA has its closest relative in *K. georgiana* (Figure 1E), a bacterium that causes clinically significant infections in humans (Sarria et al., 2001).

**FIGURE 2:**
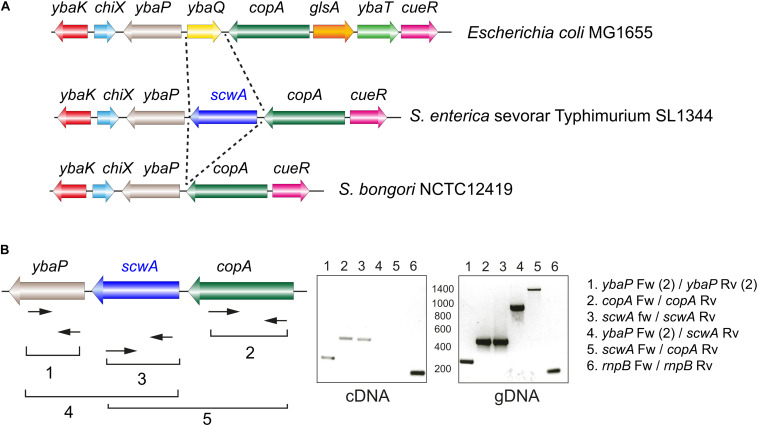
Genetic organization of the *S.* Typhimurium genomic region in which the gene *scwA* maps. **(A)** Comparison of this region in the genomes of *S.* Typhimurium strain SL1344 (GenBank entry FQ312003.1); *E. coli* strain MG1655 (GenBank entry U0096.3); and *S. bongori* strain NCTC12419 (GenBank entry LR134137.1). Note the absence of *scwA* in both *E. coli* and *S. bongori*. **(B)** Transcriptional analysis of the region with the indicated oligonucleotides that were used for RT-PCR (cDNA synthesis) and PCR reactions. The *rnpB* Fw-*rnpB* Rv product (numbered 6) correspond to the *rnp* gene mapping elsewhere and used as control. These assays were repeated with total RNA isolated in two independent biological replicates.

The *scwA* gene maps in the *S.* Typhimurium genome between *ybaP*, encoding a hypothetical protein, and *copA*, encoding a predicted copper-transporting ATPase (Figure 2A). RT-PCR assays showed that *scwA* transcription is independent from that of *ybaP* and *copA* (Figure 2B). *scwA* is replaced in the genome of *E. coli* reference strain MG1655 genome by the unrelated gene *ybaQ*, encoding an uncharacterized HTH-type transcriptional regulator. *scwA* is also absent in the genome of the *S. bongori* reference strain NCTC12419 (Figure 2A). *scwA* has a 43.1% G+C content, significantly lower than the 52.2% G+G average of the *S.* Typhimurium genome. Taken together, these data indicated that *scwA* was acquired by *S. enterica* after its divergence from *E. coli* and the non-pathogenic *S. bongori* species. The presence of ortholog proteins with rather low identity (lower than 50%) in a few clinical isolates of *E. coli* and *S. bongori*, as well as in other enteric bacteria, supports paralleled acquisition from varied sources or accelerated evolution occurring in the distinct genetic backgrounds.

### ScwA Is a Periplasmic Protein but Has No Role in Lysozyme Resistance

**FIGURE 3:**
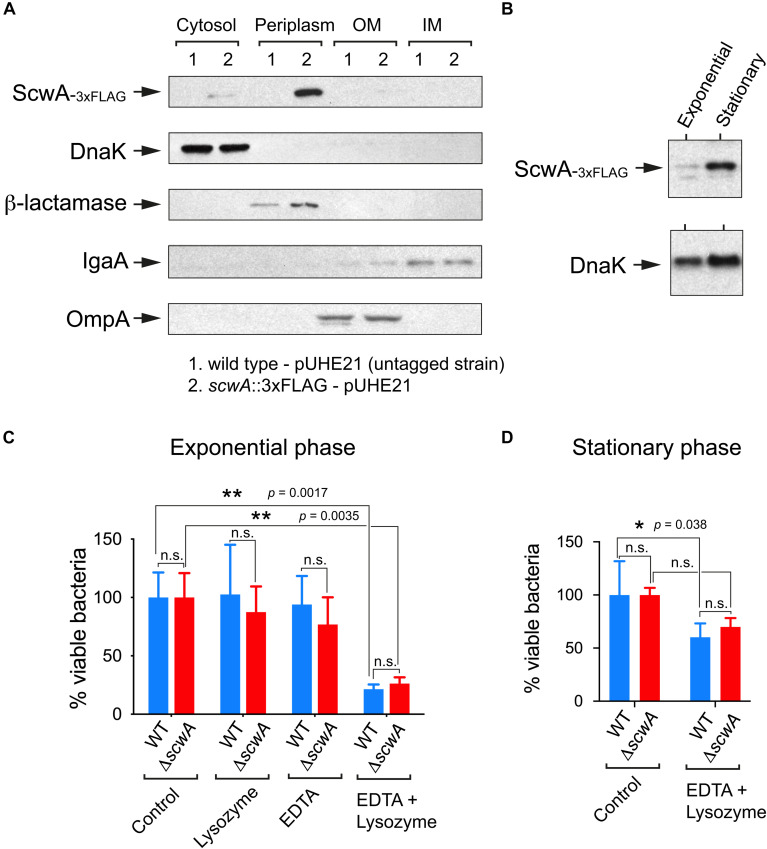
ScwA is a *S.* Typhimurium periplasmic protein with no role in lysozyme resistance. **(A)** Immunoblot analyses of subcellular fractions -cytosol, periplasm, inner membrane (IM) and outer membrane (OM)-, performed to determine ScwA location. Controls included: DnaK (cytosol); beta-lactamase (periplasm), IgaA (IM) and OmpA (OM). The *S.* Typhimurium strains used in these assays included the untagged wild-type and isogenic *scwA*::3×FLAG strains, both harboring the pUHE21 plasmid vector encoding beta-lactamase; **(B)** ScwA levels detected in whole cell lysates of actively growing (exponential) and resting (stationary phase) cultures of the chromosomally tagged *S.* Typhimurium strain *scwA*::3×FLAG. DnaK was detected as loading control. Shown are immunoblots with anti-FLAG and anti-DnaK as primary antibodies. **(C)** Lysozyme susceptibility tested in the absence/presence of the outer-membrane disrupting agent EDTA. Actively growing bacteria (OD_600_ ∼ 0.2–0.3) were exposed to these agents for 60 min before plating. **(D)** Viability assay performed in bacteria that reached stationary phase (OD_600_ ∼ 2.5) and exposed to lysozyme/EDTA for 60 min. Data are shown as mean and standard deviation of a total of four biological replicates. *p < 0.05; **p < 0.005; n.s.: not significant (two-way ANOVA with Tukey’s post-test).

To analyze ScwA production in *S.* Typhimurium, we engineered a strain bearing a 3×FLAG tag in the *scwA* 3’end and in its native chromosomal location. Immunoblot assays in subcellular fractions obtained from the *scwA*::3×FLAG tagged strain showed that ScwA localizes in the periplasm (Figure 3A). This location is consistent with the absence of hydrophobic transmembrane regions in its sequence. Production of ScwA was also seen to increase in bacteria that reached stationary phase (Figure 3B). Based on the periplasmic location of ScwA, we next tested whether the LprI domain predicted in the 185–271 amino acid region (Figure 1A) could confer lysozyme resistance. To this aim, a Δ*scwA* null mutant was compared to wild type bacteria for lysozyme susceptibility in the presence/absence of EDTA, an agent that permeabilizes the outer membrane facilitating lysozyme access to the periplasm. No differences were observed in lysozyme sensitivity between the two strains and the conditions used, neither in exponential nor in stationary phase (Figures 3C,D). Lysozyme susceptibility was not tested in conditions of ScwA overproduction as we observed that increased ScwA levels for a prolonged period of time (>60 min) led to cell lysis (Supplementary Figure 3). Altogether, the data obtained with the Δ*scwA* mutant indicated that, unlike the LprI lipoprotein of *M. tuberculosis*, ScwA does not inhibit lysozyme activity.

### ScwA Has Two Disulfide Bridges Important for Its Stability and Is Substrate of the DsbA/DsbB System

**FIGURE 4:**
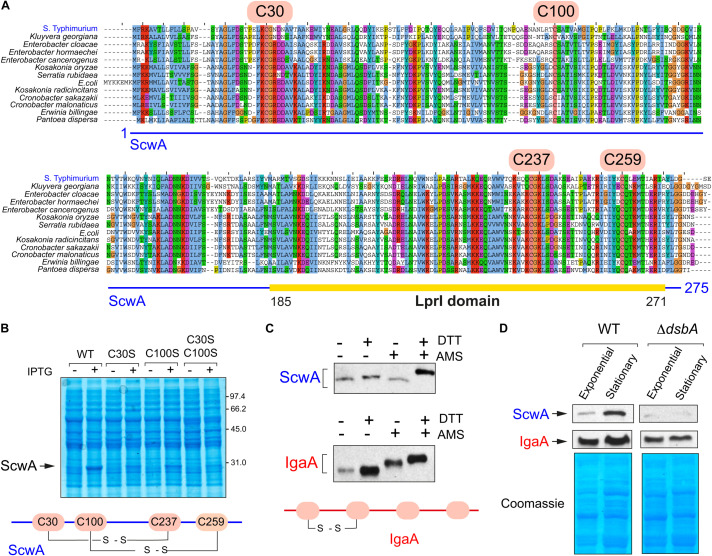
ScwA has two disulfide bonds important for protein stability. **(A)** Alignment of ScwA and ortholog proteins identified in other enteric bacteria (see also Figure 1C) that shows the presence of four conserved cysteines. In ScwA, these residues correspond to C30, C100, C237, and C259; **(B)** Expression of wild type ScwA and variants with mutated cysteines (C30S, C100S or C30S-C100S) show distinct stability following overproduced from plasmid. The IPTG concentrations used to induce expression were: 50 μM (WT); 500 μM (C30S, C100S); or 1 mM (double mutant C30S-C100S). Shown is a Coomassie stained gel of whole cell lysates obtained from bacteria grown to exponential phase (OD_600_∼0.2–0.3) and incubated with IPTG for 90 min; **(C)** Alkylation assay in membrane extracts of the *scwA*::3×FLAG-tagged strain grown to stationary phase (OD_600_∼2.5) using the cross-linker reagent 4’-acetamido-4’-maleimidylstilbene-2,2’-disulfonic acid (AMS) followed by addition (or not) of the reducing agent DTT. Note that ScwA does not change in electrophoretic mobility in the samples with no DTT regardless of the addition of AMS. Alkylation was also examined in parallel for the inner membrane IgaA, which only forms one disulfide bridge despite having four cysteines facing the periplasm (Pucciarelli et al., 2017); shown are the anti-FLAG (upper panel) and anti-IgaA (lower) immunoblots. **(D)** ScwA levels detected in whole cell lysates of the *S.* Typhimurium *scwA*::3×FLAG-tagged strain grown to exponential (OD_600_∼0.2–0.3) and stationary (OD_600_∼2.5) phases in wild type and Δ*dsbA* genetic backgrounds. Upper images are the anti-FLAG and anti-IgaA western blots, the lower panel corresponds to the Coomassie-stained gel of the same set of samples.

The Pfam domain database shows that all members of the protein family bearing the LprI domain have conserved cysteine residues^4^. The alignment of ScwA to orthologs found in enteric bacteria confirmed the presence of four conserved cysteines (Figure 4A). In ScwA, these cysteines are C30, C100, C237, and C259. To our knowledge, no functional analyses has been made on the biological significance of these conserved cysteines in any member of the LprI protein family. To investigate this, we run the DiaNNA software^5^ to assess the presence of putative disulfide bonds in ScwA. This software predicted with a high score a C30-C237 bridge (score 0.99787) and a second less probable C100-C259 bridge (score 0.03453). To assess the relevance of these putative disulfide bridges, we generated two ScwA variants, C30S and C100S. If the prediction for the C30-C237 and C100-C259 disulfide bridges was correct, the C30S and C100S variants should lack one of the two bridges. A double variant bearing C30S-C100S mutations, was also constructed. We noticed that the three variants were less stable than wild type ScwA when expressed from an expression vector, even when using high concentrations of the IPTG inducer (Figure 4B). These findings indicated that the conserved cysteines of ScwA are relevant for its stability and, as consequence, for the function of this periplasmic protein.

To further prove the existence of disulfide bridges in ScwA, we performed *in vivo* alkylation assays with the cross-linker reagent 4’-acetamido-4’-maleimidylstilbene-2,2’-disulfonic acid (AMS), running in parallel as control the inner membrane protein IgaA known to have a disulfide bridge (Pucciarelli et al., 2017). These assays showed that ScwA has two disulfide bonds since its electrophoretic mobility remained unchanged when comparing samples with/without cross-linker in the absence of the reducing agent DTT (Figure 4C). To further analyze whether these disulfide bridges stabilize ScwA, we generated a Δ*dsbA* mutant. The DsbA/DsbB system catalyzes disulfide bond formation in the oxidant periplasmic environment whereas a second system, that involving DsbC/DsbD, catalyzes disulfide bond isomerization (Goemans et al., 2014). Several DsbA functional paralogues, DsbL and SrgA, also exist in *S.* Typhimurium (Heras et al., 2010). The lack of functional DsbA was, however, sufficient to compromise ScwA stability, with much less amount of the protein detected in the Δ*dsbA* mutant (Figure 4D). Such effect was noticed in bacteria growing to exponential and stationary phases (Figure 4D). Taken together, these findings showed that ScwA requires the oxidoreductase DsbA for its stability in the periplasm.

### ScwA Controls the Levels of the Murein Lytic Transglycosylase MltD

**FIGURE 5:**
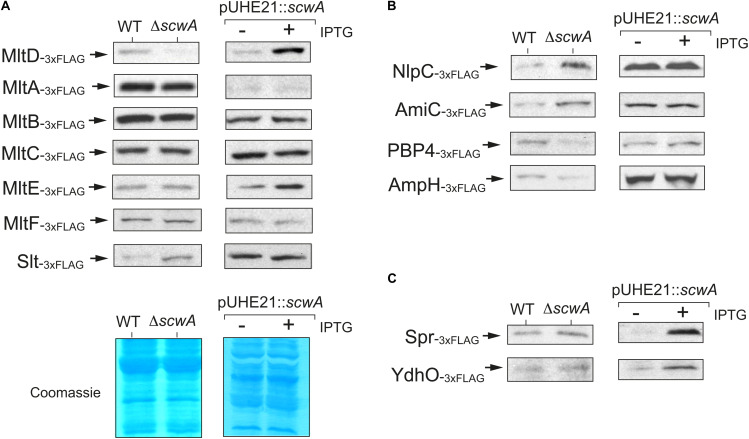
*S.* Typhimurium ScwA controls the levels of the murein lytic transglycosylase (LT) MltD and a few other PG hydrolases. **(A)** Immunoblot assays with anti-FLAG antibody of whole cell lysates showing positive correlation between ScwA and MltD levels, these later decreasing in the absence of ScwA but increasing in response to the overproduction of ScwA from plasmid pUHE21. Among the other LTs tested, MltA, MltB, MltC, MltE (EmtA), MltF or Slt, only a minor effect is seen in the case of Slt. The comparison between wild type and Δ*scwA* strains (left columns) was performed in stationary phase cultures (OD_600_∼2.5) whereas production of ScwA from the pUHE21 plasmid was induced in exponential phase cultures (OD_600_∼0.2–0.3) with 1 mM IPTG for 40 min. The lower image shows representative Coomassie-stained gels demonstrating proper samples adjustment in the two types of assays designed to test the effect due to either the absence or overproduction of ScwA. **(B)** Altered levels of NlpC, AmiC, PBP4, and AmpH observed in the Δ*scwA* mutant. None of these proteins change, however, in their relative amounts in response to ScwA overproduction (right columns). Shown are anti-FLAG immunoblots of whole cell lysates. The left (wt and Δ*scwA* strains) and right (–/+ induction of ScwA expression with IPTG) samples correspond to the same conditions as described in panel A (stationary and exponential phase, respectively). **(C)** Increased levels of the D,D-endopeptidases Spr (MepS) and YdhO (MepH) in response to ScwA overproduction. Unlike MltD, these two enzymes remain unaltered when comparing wild type and Δ*scwA* strains. The respective 3×FLAG-tagged strains used in these assays are described in Supplementary Table 1.

The absence of a phenotype related to lysozyme resistance in bacteria lacking ScwA (Figures 3C,D) and the cell lysis triggered by prolonged overproduction of ScwA (Supplementary Figure 3), prompted us to seek whether this protein could influence the activity of periplasmic enzymes acting on the PG. To test this hypothesis, we generated a collection of 34 isogenic *S.* Typhimurium mutants in which the genes encoding periplasmic enzymes involved in PG metabolism were 3×FLAG-tagged at their 3’ ends in their native chromosomal locations. A parallel series was generated in the Δ*scwA* genetic background. The tagged PG enzymes include: (i) PBPs, both the biosynthetic and those with carboxy- or endopeptidase activity in stem peptides of the PG; (ii) L,D-transpeptidases; (iii) the beta-lactam insensitive glycosyltransferase MtgA; (iv) D,D- and D,L-endopeptidases; (v) amidases that cleave the N-acetyl-muramic acid (MurNAc)-L-alanine amide bond; and (vi) murein lytic transglycosylases (LT). Immunoblot assays showed that the absence of ScwA affected predominantly the levels of the murein transglycosylase MltD (Figure 5A). The opposite was also observed when overproducing ScwA for only a short period of time, 40 min, in which no cell lysis is noticeable (Supplementary Figure 3). In this later case, ScwA overproduction resulted in concomitant increase of MltD levels (Figure 5A).

Apart from MltD, no such correlation was observed between ScwA levels and those of other related LTs such as MltA, MltB, MltC, MltE (EmtA), MltF or the soluble LT named Slt. However, a subtle increase in Slt levels was noted in the absence of ScwA (Figure 5A). Although at a lower extent than for MltD, the absence of ScwA also altered the levels of a few additional enzymes involved in PG metabolism: (i) NlpC and PBP4 (DacB), with D,D-endopeptidase activity on the D-Ala-*meso*-Dap bridges; (ii) the amidase AmiC; and (iii) the D,D-carboxypeptidase AmpH, which cleaves the terminal D-Ala-D-Ala of the stem peptide as PBP4 does (Figure 5B). Two other PG hydrolases, the D,D-endopeptidase YdhO (MepH) and the L,D-carboxypeptidase Spr (MepS); increased their relative levels but only after a short period (40 min) of ScwA overproduction (Figure 5C). We did not observe significant changes in protein levels when testing an additional set of 21 enzymes involved in peptidoglycan metabolism, including L,D-transpeptidases, PBPs of high and low molecular weight, enzymes involved in the recycling pathway or the glycosyltransferase MtgA (Supplementary Figure 4).

**FIGURE 6:**
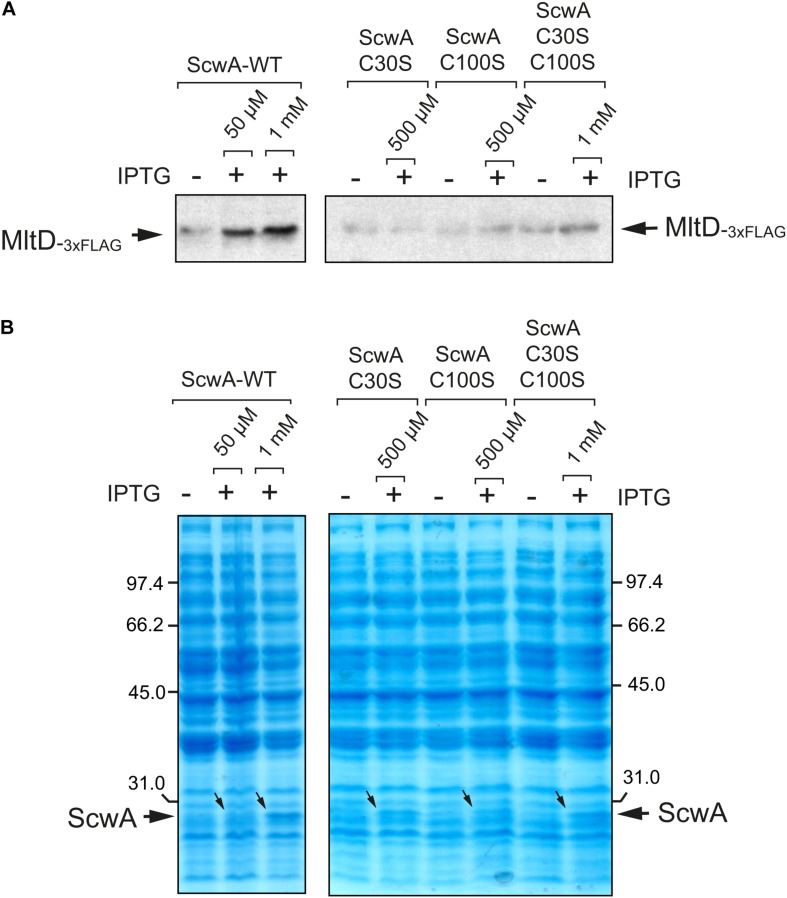
The ScwA variants lacking disulfide bridges loss the capacity to modulate the levels of the lytic transglycosylase MltD. **(A)** Immunoblots obtained with anti-FLAG antibodies using whole cell lysates of isogenic Δ*scwA mltD*::3×FLAG strains expressing from plasmid either wild type ScwA or variants lacking disulfide bridges -C30S, C100S, C30S/C100S grown to exponential phase (OD_600_∼0.2–0.3). The IPTG inducer was used at the indicated concentrations for 90 min, with the only exception of wild type ScwA 1 mM IPTG, condition maintained for 40 min to prevent cell lysis. Increase in relative levels of MltD occur exclusively when producing wild type ScwA after the addition of either 50 μM or 1 mM of IPTG. **(B)** Coomassie staining of the whole cell lysates samples shown in panel A, confirming the production of the distinct ScwA versions used. Note that although the increase of wild type ScwA obtained with 50 μM IPTG are barely seen, it is sufficient to see increase MltD levels, an effect not seen with any of the ScwA variants lacking disulfide bridges.

Given the clear effect of ScwA over MltD levels, both in physiological conditions and following overproduction (Figure 5A), we next tested whether the putative disulfide bridges important for ScwA stability (Figure 4B) are relevant for this regulation. To this aim, wild type ScwA and the C30S, C100S and C30S/C100S variants were expressed in a Δ*scwA* mutant bearing the *mltD*::3×FLAG tagged allele. These assays showed that MltD relative levels increased only after expressing wild type ScwA, even in low amounts following induction with 50 μM IPTG (Figures 6A,B). These data confirmed the requirement of disulfide bridges for the regulation that ScwA exerts over MltD.

Taking together, the data obtained with the collection of strains bearing tagged PG enzymes supported ScwA as regulatory factor influencing in the periplasm the levels of a reduced number of hydrolytic enzymes that cleave distinct bonds in the PG meshwork.

### ScwA Is Downregulated by *S.* Typhimurium Inside Eukaryotic Cells but Required to Tone Down Virulence

**FIGURE 7:**
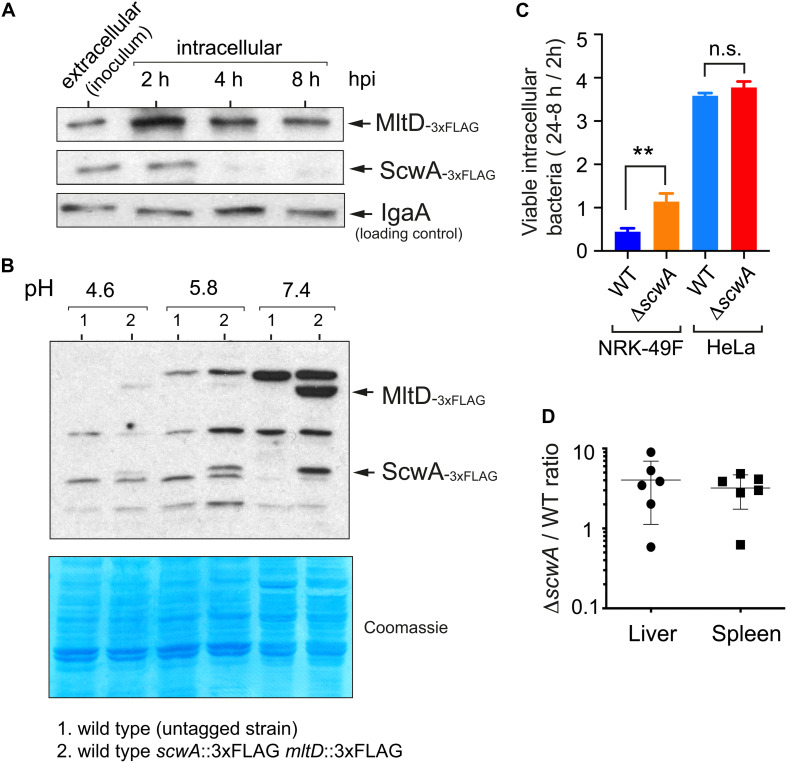
ScwA limits pathogenicity in the mouse infection model despite displaying reduced expression by intracellular bacteria residing in acidic phagosomes. **(A)** ScwA levels decreases as the intracellular infection progresses in the NRK-49F rat fibroblast model. Whole cell extracts of infected fibroblasts were obtained at the indicated post-infection times as described (Nunez-Hernandez et al., 2013). Extracellular bacteria correspond to stationary phase cultures obtained in non-shaking conditions, which favors host cell invasion. The doubly tagged strain used in these assays was *scwA*::3×FLAG *mltD*::3×FLAG, the immunoblots shown were obtained with anti-FLAG and anti-IgaA as primary antibodies, as indicated. **(B)** ScwA production is strongly repressed as bacteria grow in acidified media. Shown are whole cell lysates obtained from bacteria grown to stationary phase in PCN minimal medium adjusted at the indicated pH. Note the decrease in MltD levels as the pH drops, concomitant with ScwA. The bacteria used is the doubly tagged *scwA*::3×FLAG *mltD*::3×FLAG strain. Lower panel corresponds to a Coomassie-stained gel of the same samples. **(C)** Proliferation rate of wild type and Δ*scwA* strains inside NRK-49F fibroblasts and HeLa epithelial cells. Ratios of viable intracellular bacteria are 24 vs. 2 h post-infection (NRK-49F) and 8 h vs. 2 hpi (HeLa). Data are shown as mean and standard deviation of a total of three biological replicates. **p < 0.005; n.s.: not significant (*t*-test); **(D)** Competition assay in 8-week old female BALB/c mice performed as described (Dominguez-Bernal et al., 2004). Colonies of wild type and Δ*scwA*::.Km^R^ bacteria were quantified in liver and spleen extracts at 2 days post-challenge. Shown are the ratios between mutant and wild type bacteria per individual mouse and for each organ.

Our previous studies show that enzymes involved in *S.* Typhimurium PG metabolism that are absent in *E. coli* like EcgA, PBP2_SAL_, and PBP3_SAL_, respond to environmental cues found by the pathogen inside the eukaryotic phagosome as acidic pH and nutrient limitation (Rico-Perez et al., 2016; Castanheira et al., 2017, 2018, 2020). Since ScwA is also absent in non-pathogenic *E. coli* and modulates levels of a subset of enzymes acting on the PG, we reasoned that it could play a role in virulence. We first examined ScwA relative levels in intracellular *S.* Typhimurium after entry into cultured fibroblasts, a host cell type in which EcgA, PBP2_SAL_, and PBP3_SAL_ are strongly up-regulated by intracellular bacteria and in which the pathogen establishes a persistent infection that has been characterized extensively (Nunez-Hernandez et al., 2013, 2014; López-Montero et al., 2016). Infections were performed with a doubly tagged *mltD*::3×FLAG *scwA*::3×FLAG strain. These assays revealed that ScwA is down-regulated as the pathogen establishes the persistence state, a time estimated to start at 4 h post-infection by earlier studies (Figure 7A; Nunez-Hernandez et al., 2013, 2014). Interestingly, MltD levels also decrease in intracellular bacteria along the time, although this hydrolase remains visible even at late post-infection times, 8 h (Figure 7A). This result suggested that ScwA could be more labile than MltD once intracellular bacteria arrest ScwA production. The lack of evidence for *de novo* synthesis of ScwA by intracellular *S.* Typhimurium raised the possibility that unlike EcgA, PBP2_SAL_, and PBP3_SAL_, the production of ScwA could be repressed by intra-phagosomal signals such as acid pH. Growth of *S.* Typhimurium in minimal media adjusted to varied pH values (4.6, 5.8, and 7.4) confirmed such hypothesis with decreasing amounts of ScwA as the pH value dropped and no visualization of the protein at pH 4.6 (Figure 7B). This result was consistent with the data obtained in intracellular persistent bacteria (Figure 7A). Interestingly, MltD levels correlated to those of ScwA at all pH values tested, with the highest amounts registered at neutral pH (Figure 7B). Such correlation confirmed the functional link between the two proteins.

Since PG metabolism plays a central role in infection, we next examined whether ScwA contributes to *S.* Typhimurium pathogenicity. In comparison to wild type bacteria, the Δ*scwA* mutant exhibited higher proliferation rate inside cultured fibroblasts (Figure 7C) and enhanced virulence in the BALB/c murine typhoid model (Figure 7D). Since the presence of ScwA modulates relative levels of PG enzymes like MltD, NlpC, AmiC, PBP4 (DacB), and AmpH (Figures 5A,B), we sought to determine whether altered expression of any of this group of enzymes was responsible for the hypervirulent phenotype exhibited by the Δ*scwA* mutant. Competition experiments in BALB/c mice using the respective double mutants did not show statistical differences in their capacity to colonize target organs, liver and spleen, compared to the single Δ*scwA* mutant. All the mutants tested displayed higher virulence than wild type bacteria (Supplementary Figure 5). Collectively, these data suggested that ScwA modulates the course of the infection by controlling the activity of a subset of enzymes involved in PG remodeling. In the case of those PG enzymes with lower levels in the Δ*scwA* mutant (MltD, and PBP4 (DacB) / AmpH at lesser extent) (Figure 5), the loss of these enzymes in the respective double mutants did not exacerbate the hypervirulent phenotype (Supplementary Figure 4). We therefore concluded that individual inactivation of enzymes among those of the group controlled by ScwA is not sufficient to alter significatively the hypervirulence shown by the Δ*scwA* mutant.

## Discussion

In this study, we characterized a novel periplasmic protein in *S.* Typhimurium, ScwA, which is absent in most isolates of its closest phylogenetic relative bacterium, *E. coli*. The *in silico* analysis on ScwA showed that the closest ortholog, identified in the opportunistic pathogen *Kluyvera georgiana*, is highly similar in size (∼275 aa) and accounts for a 50% identity uniformly spread along the protein. These features, similar protein size and identity along the entire protein, were observed in the rest of orthologs found in distinct enteric bacteria with identity decreasing up to 35%. Other remarkable features found in this set of proteins is that they are produced exclusively by bacterial pathogens, display four highly conserved cysteine residues, and that all bear a LprI domain in the C-terminal region of the protein, annotated in Pfam database as “Lysozyme inhibitor” (Pfam PF07007) but with yet no experimental evidence sustaining such a role. Indeed, we were unable to find such functional relationship for ScwA, suggesting that this LprI domain could play another biological role. Importantly, the lipoprotein LprI of *M. tuberculosis*, in which the LprI domain was first reported (Sethi et al., 2016), bears a second domain named MliC (Pfam PF09864), which has been experimentally proved to be sufficient for conferring lysozyme resistance. Most periplasmic proteins implicated in lysozyme resistance described in Gram-negative bacteria, all belonging to the Proteobacteria phylum, are of small size, in the range of 100–150 residues, and bear exclusively the MliC domain. Some examples of these lysozyme inhibitors are Ivy and PliG from *E. coli* (Vanderkelen et al., 2011; Liu et al., 2015), and PliC from *S.* Enteritidis, *S.* Typhimurium and *Pseudomonas aeruginosa* (Callewaert et al., 2012). Another difference between ScwA and these lysozyme inhibitors, including LprI from *M. tuberculosis*, is that is not anchored to the membrane whereas bona fide lysozyme inhibitors are canonical lipoproteins with an N-terminal sequence cleaved by the lipoprotein-specific signal peptidase.

ScwA, which is free in the periplasm and has the LprI domain but lacks the MliC domain, might have been not specialized in the defense against exogenous enzymes but, instead, in regulating stability of endogenous enzymes involved in PG metabolism. Such control could be driven by a particular folding state in ScwA mediated by the two disulfide bridges that our experimental data supports with a putative C30-C237 and C100-C259 configuration. This distribution spans across part of the LprI domain (residues 185–271), suggesting that such hypothetical domain could play a key role in function. The fact that overexpression of ScwA variants lacking the disulfide bridges did not lead to increased MltD levels sustains this idea of a critical role of these disulfide bridges for proper folding of ScwA to perform its regulatory function.

Our next study focused on the identification of PG enzymes potentially related with ScwA at the functional level. This comprehensive analysis uncovered a positive regulation of MltD. The biological function of MltD, which has a “soluble lytic transglycosylase” (Slt) domain and two LysM domains involved in PG binding (Bateman and Bycroft, 2000), is still poorly understood. Interestingly, none of the other LT known in *E. coli* and *S.* Typhimurium -MltA, MltB, MltC, MltE (EmtA), MltF, or Slt- have a similar domain architecture. Among these LTs, MltD is the only one bearing LysM domains, for which the structure at the atomic level was resolved, consisting in two helices packing on the same side of an anti-parallel beta-sheet (Bateman and Bycroft, 2000). These unique structural features of MltD could explain the exquisite regulation mediated by ScwA, not reflected in other similar membrane-bound LTs.

There are also studies in *E. coli* that reported regulation of the *mltD* transcript by RNAse III (Lim et al., 2014). Our data obtained at the protein level also show that the pH is a factor affecting MltD levels, concretely less protein as the pH value becomes more acidic. This regulation is also visible in intracellular *S.* Typhimurium, with slightly decreasing MltD levels that may even correspond to the protein brought by the invading bacteria, therefore discarding *de novo* synthesis in the acidic intra-phagosomal environment. Future work should assess whether this RNAse III-mediated regulation and that exerted by ScwA are interconnected.

It is rather coincidental that ScwA, which our data support capable of modulating MltD levels is, however, not able to confer lysozyme resistance considering than both enzymes, MltD and lysozyme, cleave the same β(1–4) glycosidic bond between N-acetyl-muramic acid (MurNAc) and N-acetyl-glucosamine (GlcNAc). Our interpretation to these findings is that inhibition of lysozyme activity may be sustained mainly by the MliC domain (absent in ScwA) whereas the LprI domain may facilitate binding to specific features of MltD structure, for example the C-half region of the protein harboring the LysM domains. Future studies with purified proteins and variants lacking specific regions of both ScwA and MltD, especially regarding the LrpI and LysM domains, might provide insights into a putative protein-protein interaction occurring in the periplasm.

Our data also showed that ScwA modulates *S.* Typhimurium virulence, more especially attenuating host damage since the lack of ScwA translated in higher bacterial loads in target organs. There are precedents of functions encoded exclusively in genomes of bacterial pathogens that, intriguingly, are also involved in reducing host damage. In *S.* Typhimurium, the absence of PcgL, a D-Ala-D-Ala dipeptidase or the regulator PmrA, which controls modifications in the lipopolysaccharide (LPS), render bacteria hypervirulent (Mouslim et al., 2002; Choi and Groisman, 2013). Another illustrative example is the loss of a protein named Cj0371 in *Campylobacter jejuni*, whose deficiency translates in hypermotility, enhanced chemotaxis and increased host colonization (Du et al., 2016). In *M. tuberculosis* the lack of PknE, a serine/threonine kinase required for several adaptive responses, leads to enhance virulence in a guinea pig model (Kumar et al., 2013). The loss in cyclopropanation of mycolic acids bound to trehalose in this pathogen also results in a hypervirulent phenotype (Riley, 2006). Most of these cases are linked to modifications in the cell envelope of the invading pathogen, which reinforces the importance of cell wall homeostasis during infection.

How could ScwA modulate simultaneously MltD activity as well as that of a reduced set of other PG enzymes such as Slt, NlpC, AmiC, PBP4 (DacB), and AmpH, to adjust virulence for successful host colonization? Our data clearly establish positive and negative correlations between the relative levels of ScwA and those of some PG enzymes and it is precisely this balance what might be required during the infection. The ultimate outcome of such regulation over a few hydrolases could be to minimize the release of proinflammatory PG fragments in host tissues. This strategy might be widely used by *Salmonella*, a pathogen prone to establish chronic and asymptomatic infections. In contrast, other pathogens like *Neisseria gonorrhoeae* causes infections that progress with high levels of proinflammatory molecules. *N. gonorrhoeae* uses the lytic transglycosylases LtgA and LtgD in its interaction with the host, resulting in the release of PG fragments that promote gonococcal infections by over-stimulation of host immune defenses (Knilans et al., 2017; Ragland et al., 2017).

In summary, our study emphasizes how the fine regulation of enzymes acting on PG metabolism is critical for *S.* Typhimurium to progress in the infection. In this case, we identified ScwA as a periplasmic protein that could contribute to maintenance of cell wall homeostasis during host colonization by tuning down the activity of a subset of degradative PG enzymes.

## Author’s Note

Accumulating evidence supports readjustment of peptidoglycan metabolism in bacterial pathogens in response to host cues. Some of these changes involve accessory enzymes that act specifically during the infection process and are absent in non-pathogenic bacteria. Our study in the intracellular bacterial pathogen *Salmonella enterica* serovar Typhimurium demonstrates that pathogen-specific functions can also regulate the levels of “core enzymes” that remodel peptidoglycan structure. This control might be relevant for fine-tuning the course of the infection, for example by decreasing the release of proinflammatory fragments derived from peptidoglycan metabolism and, in this manner, attenuating the level of damage inflicted to the host. From the pathogen side, such strategy may favor the establishment of persistent infections, facilitating a long-lasting residence in the host.

## Data Availability Statement

The original contributions presented in the study are included in the article/Supplementary Material, further inquiries can be directed to the corresponding author.

## Ethics Statement

The animal study was reviewed and approved by the Environment Council (Consejería de Medio Ambiente) of the Regional Government of Madrid, under license PROEX 110/19.

## Author Contributions

JC, SC, MP, and FG-P contributed to the study design. JC and MP collected and analysed the data that were further discussed with FG-P. JC, MP, and FG-P interpreted the data. The first draft was written by FG-P, which was critically commented by the rest of authors. All authors approved the final version of the manuscript.

## Conflict of Interest

The authors declare that the research was conducted in the absence of any commercial or financial relationships that could be construed as a potential conflict of interest.

## Footnotes

1 http://bioinf.cs.ucl.ac.uk/psipred/

2 http://www.sbg.bio.ic.ac.uk/~phyre2

3 http://smart.embl-heidelberg.de/

4 https://pfam.xfam.org/family/lprI

5 http://clavius.bc.edu/~clotelab/DiANNA/
