# Supplementary material for: A Novel Salmonella Periplasmic Protein Controlling Cell Wall Homeostasis and Virulence
Source: Front Microbiol. 2021 Feb 19;12:633701. doi: 10.3389/fmicb.2021.633701 (PMC7933661; doi:10.3389/fmicb.2021.633701)
Supplement: Supplementary file 1 [file Data_Sheet_1.PDF]

Supplementary Material

A novel *Salmonella* periplasmic protein controlling cell wall homeostasis and virulence

Juan J. **Cestero**, Sónia **Castanheira**, M. Graciela **Pucciarelli** and Francisco **García-del Portillo**

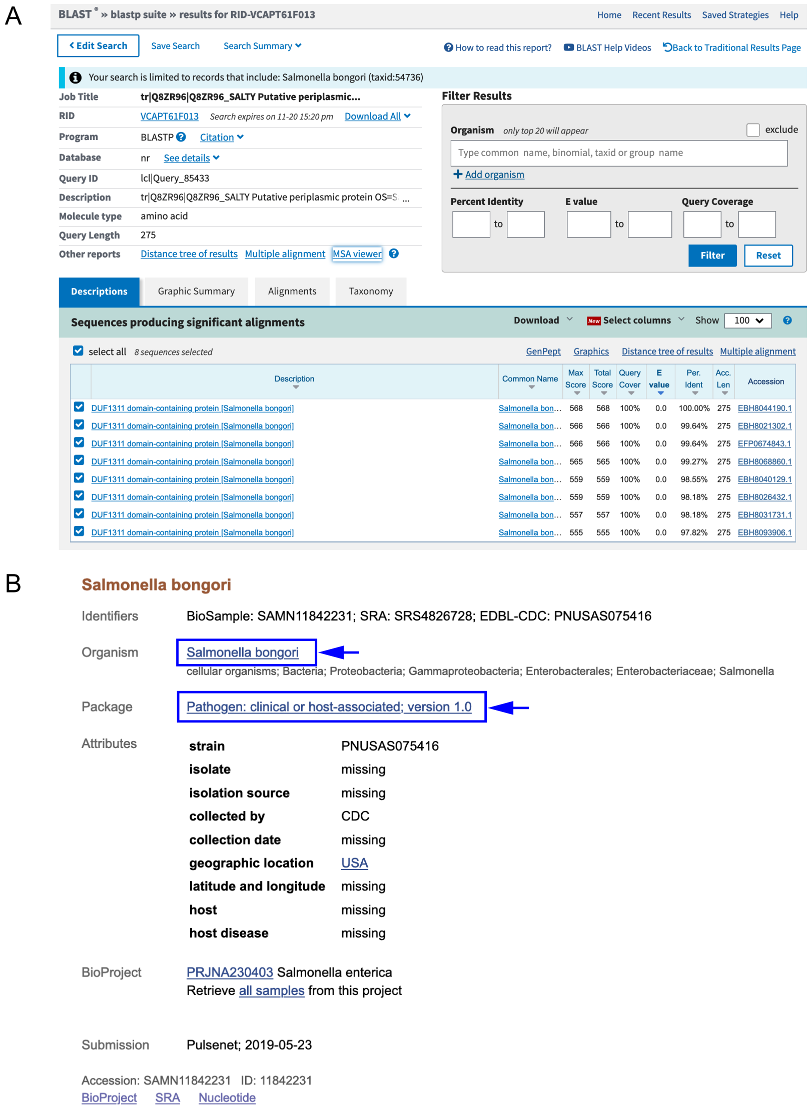

Figure S1. BLASTP identifies *S.* Typhimurium ScwA (SL1344_0490) orthologs in a few clinical or host-associated isolates of *S. bongori*. (A) List of the only eight hits obtained after searching in all *S. bongori* genomes deposited in databases; (B) Example of the information of one of these eight isolates referring as clinical or host-associated. The other seven isolates have all the same description regarding their respective sources.

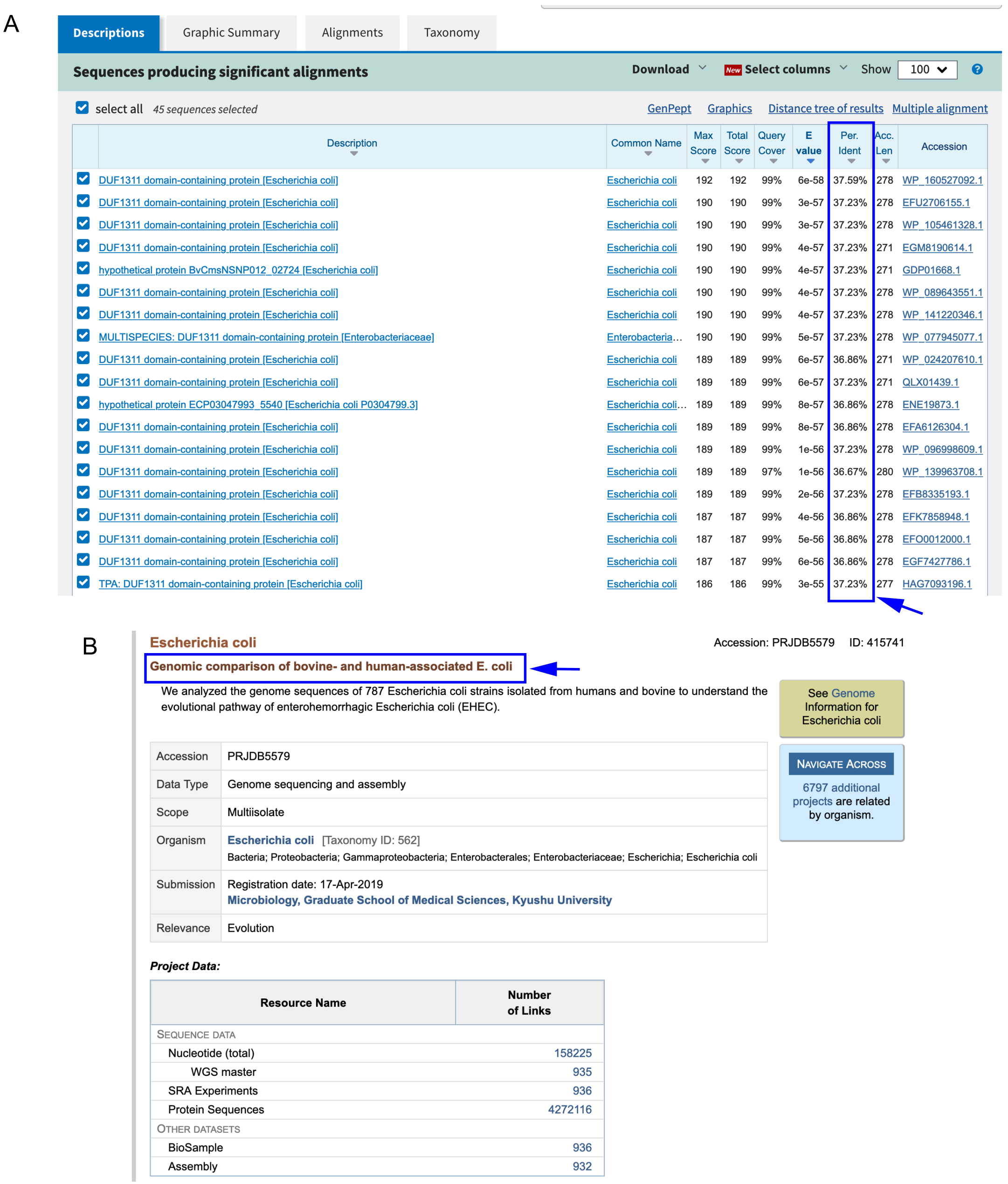

A

Descriptions | Graphic Summary | Alignments | Taxonomy

Sequences producing significant alignments

Download | New Select columns | Show 100

select all *45 sequences selected*

GenPept | Graphics | Distance tree of results | Multiple alignment

Description	Common Name	Max Score	Total Score	Query Cover	E value	Per. Ident	Acc. Len	Accession
DUF1311 domain-containing protein [Escherichia coli]	Escherichia coli	192	192	99%	6e-58	37.59%	278	WP_160527092.1
DUF1311 domain-containing protein [Escherichia coli]	Escherichia coli	190	190	99%	3e-57	37.23%	278	EFU2706155.1
DUF1311 domain-containing protein [Escherichia coli]	Escherichia coli	190	190	99%	3e-57	37.23%	278	WP_105461328.1
DUF1311 domain-containing protein [Escherichia coli]	Escherichia coli	190	190	99%	4e-57	37.23%	271	EGM8190614.1
hypothetical protein BvCmsNSNP012_02724 [Escherichia coli]	Escherichia coli	190	190	99%	4e-57	37.23%	271	GDP01668.1
DUF1311 domain-containing protein [Escherichia coli]	Escherichia coli	190	190	99%	4e-57	37.23%	278	WP_089643551.1
DUF1311 domain-containing protein [Escherichia coli]	Escherichia coli	190	190	99%	4e-57	37.23%	278	WP_141220346.1
MULTISPECIES: DUF1311 domain-containing protein [Enterobacteriaceae]	Enterobacteria...	190	190	99%	5e-57	37.23%	278	WP_077945077.1
DUF1311 domain-containing protein [Escherichia coli]	Escherichia coli	189	189	99%	6e-57	36.86%	271	WP_024207610.1
DUF1311 domain-containing protein [Escherichia coli]	Escherichia coli	189	189	99%	6e-57	37.23%	271	QLX01439.1
hypothetical protein ECP03047993_5540 [Escherichia coli P0304799.3]	Escherichia coli...	189	189	99%	8e-57	36.86%	278	ENE19873.1
DUF1311 domain-containing protein [Escherichia coli]	Escherichia coli	189	189	99%	8e-57	36.86%	278	EFA6126304.1
DUF1311 domain-containing protein [Escherichia coli]	Escherichia coli	189	189	99%	1e-56	37.23%	278	WP_096998609.1
DUF1311 domain-containing protein [Escherichia coli]	Escherichia coli	189	189	97%	1e-56	36.67%	280	WP_139963708.1
DUF1311 domain-containing protein [Escherichia coli]	Escherichia coli	189	189	99%	2e-56	37.23%	278	EFB8335193.1
DUF1311 domain-containing protein [Escherichia coli]	Escherichia coli	187	187	99%	4e-56	36.86%	278	EFK7858948.1
DUF1311 domain-containing protein [Escherichia coli]	Escherichia coli	187	187	99%	5e-56	36.86%	278	EFO0012000.1
DUF1311 domain-containing protein [Escherichia coli]	Escherichia coli	187	187	99%	6e-56	36.86%	278	EGF7427786.1
TPA: DUF1311 domain-containing protein [Escherichia coli]	Escherichia coli	186	186	99%	3e-55	37.23%	277	HAG7093196.1

B

Escherichia coli

Accession: PRJDB5579 ID: 415741

Genomic comparison of bovine- and human-associated E. coli

We analyzed the genome sequences of 787 Escherichia coli strains isolated from humans and bovine to understand the evolutional pathway of enterohemorrhagic Escherichia coli (EHEC).

See Genome Information for Escherichia coli

NAVIGATE ACROSS
6797 additional projects are related by organism.

Accession	PRJDB5579
Data Type	Genome sequencing and assembly
Scope	Multiisolate
Organism	**Escherichia coli** [Taxonomy ID: 562]<br>Bacteria; Proteobacteria; Gammaproteobacteria; Enterobacterales; Enterobacteriaceae; Escherichia; Escherichia coli
Submission	Registration date: 17-Apr-2019<br>**Microbiology, Graduate School of Medical Sciences, Kyushu University**
Relevance	Evolution

Project Data:

Resource Name	Number of Links
SEQUENCE DATA	
Nucleotide (total)	158225
WGS master	935
SRA Experiments	936
Protein Sequences	4272116
OTHER DATASETS	
BioSample	936
Assembly	932

Figure S2. BLASTP identifies *S.* Typhimurium ScwA (SL1344_0490) orthologs in a few isolates of *E. coli* from food, animal and human sources. **(A)** List of 19 hits having a coverage >95%. identified after searching in all *E. coli* genomes deposited in databases. Note that the homology in stands in 36-37% in all cases (blue arrow); **(B)** Example of the information provided by one Bioproject in which the genome of some of the *E. coli* isolates with ScwA orthologs, were sequenced. Blue arrow indicates the source of isolates, bovine- and human-associated.

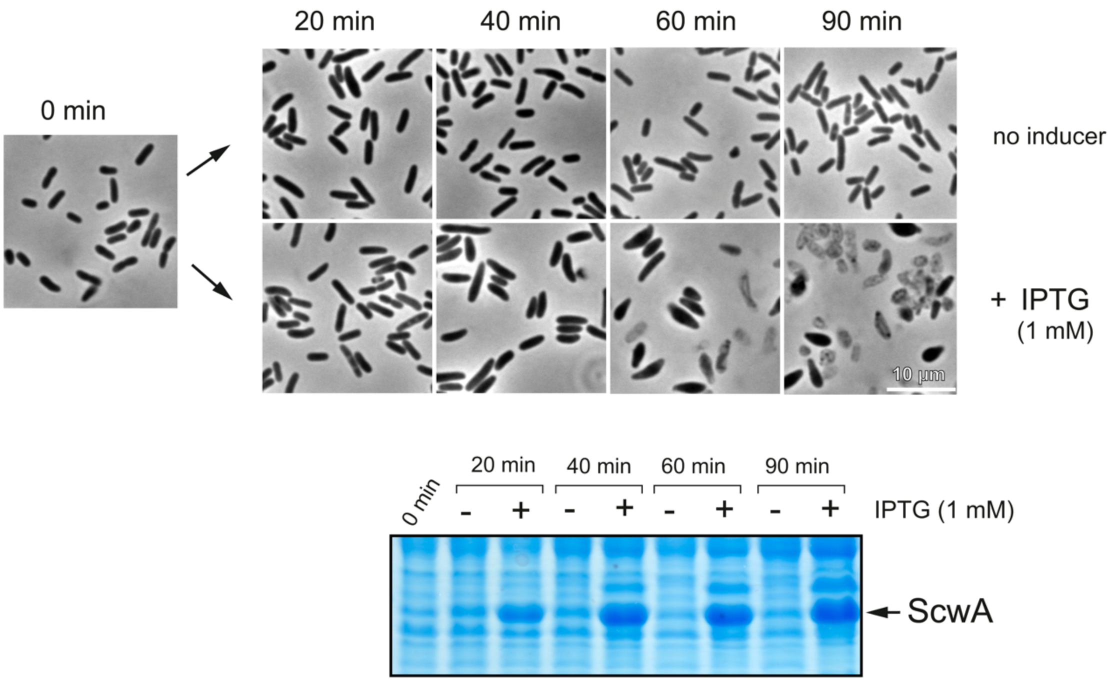

Figure S3. Sustained overproduction of ScwA results in bacterial lysis. An overnight culture of wild type strain bearing the pUHE21::*scwA* plasmid was diluted 1:100 in fresh LB medium to reach exponential phase at OD_{600}~ 0.2. At this time, the culture was divided in two, adding IPTG (1 mM) to one of them. Samples were collected 0, 20, 40, 60 and 90 min after IPTG addition, centrifuged (4300 x *g*, 5 min, RT), washed in PBS pH 7.2 and processed for either electrophoresis analysis by addition of Laemmli buffer (whole cell lysates) or fixed with 3% paraformaldehyde for microscopy analyses. Images were acquired on an inverted Leica DMI 600B microscope with an automated CTR/7000 HS controller (Leica Microsystems) and an Orca-R2 charge-coupled-device (CCD) camera (Hamamatsu Photonics). Upper images depict phase contrast microscopy images. Note the appearance of cell ghosts (PG sacculi) from 60 min after IPTG addition, suggesting imbalance in the activity of PG enzymes. Bar, 10 µm. Lower panel corresponds to the Coomassie-stained gel of the same samples. The position of ScwA in the gel is indicated.

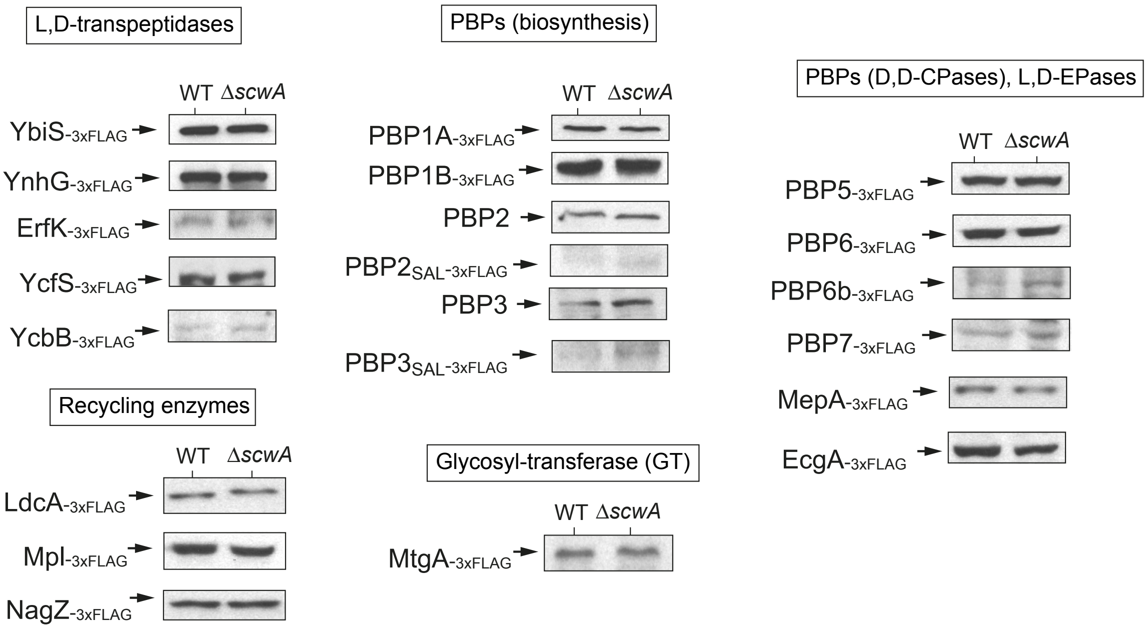

Figure S4. Levels of distinct PG enzymes in isogenic *S.* Typhimurium wild type and Δ*scwA* strains bearing 3xFLAG-tagged alleles of the respective enzymes in their native chromosomal locations (see Methods and Supplementary Tables S1 and S2, for details).

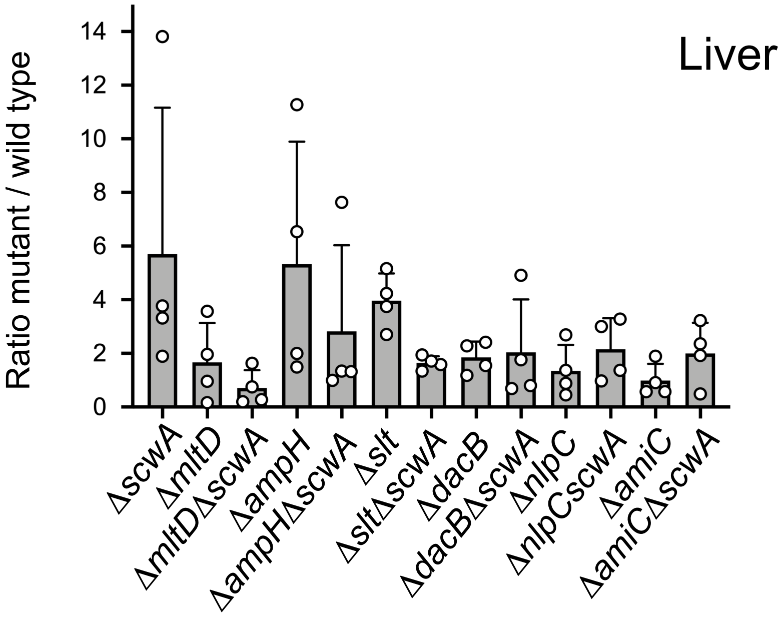

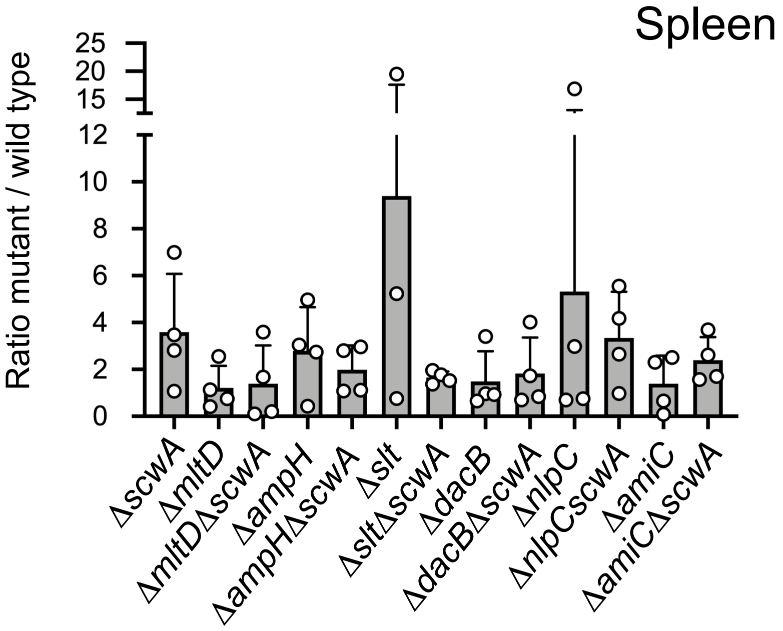

Figure S5. The hypervirulence phenotype of the Δ*scwA* strain is not suppressed by the individual loss of any of distinct PG enzymes that are functionally related to ScwA. Challenge was done intra-peritoneally in groups of four mice that were sacrificed at 48 h. Liver and spleen extracts were then obtained for counting of viable bacteria, as described (Dominguez-Bernal et al., 2004). Data were analyzed using One-way ANOVA and no significant differences were observed ($P > 0.05$).

Table S1. Bacterial strains and plasmids used in the study

Strain	Genotype	Reference
E.coli		
DH5α	*F⁻ endA1 glnV44 thi-1 recA relA1 gyrA96 deoR nupG φ80dlacZΔM15 Δ(lacZYA-argF)U169, hsdR17(rK⁻mK⁺), λ⁻*	(Hanahan, 1983)
***S.* Typhimurium**		
SV5015	SL1344 *hisG*⁺	(Vivero et al., 2008)
MD3755	SV5015 Δ*scwA*-Km	This study
MD5241	SV5015 Δ*scwA*	This study
MD3740	SV5015 *scwA*::3xFLAG-Km	This study
MD4304	SV5015 *scwA::3*xFLAG	This study
MD3581	SV5015 pUHE21	This study
MD3582	SV5015 pUHE21::*scwA*	This study
MD4346	SV5015 pUHE21::*scwA* Cys30-Ser	This study
MD4351	SV5015 pUHE21::*scwA* Cys100-Ser	This study
MD4352	SV5015 pUHE21::*scwA* Cys30-Ser Cys100-Ser	This study
MD4372	SV5015 Δ*dsbA scwA*::3xFLAG- Km	This study
MD4392	SV5015 *scwA*::3xFLAG-Km pUHE21	This study
MD5250	SV5015 *scwA*::3xFLAG *mltD*::3xFLAG-Km	This study
MD3266	SV5015 *ecgA*::3xFLAG	(Rico-Pérez et al., 2016)
MD3368	SV5015 *mrcA*::3xFLAG *dacB*::3xFLAG *dacA*::3xFLAG *STM1910*::3xFLAG *ampH*::3xFLAG-Km	This study
MD3378	SV5015 *slt*::3xFLAG *ldcA*::3xFLAG *amiC*::3xFLAG *mltA*::3xFLAG-Km	This study
MD3707	SV5015 *mrcB*::3xFLAG *dacC*::3xFLAG *STM1836*::3xFLAG *pbpG*::3xFLAG	This study
MD3712	SV5015 *nagZ*::3xFLAG *mtgA*::3xFLAG	This study
MD3790	SV5015 *mepA*::3xFLAG *dacD*::3xFLAG *mltF*::3xFLAG *mltB*::3xFLAG-Km	This study
MD3708	SV5015 *emtA*::3xFLAG *mltD*::3xFLAG *mltC*::3xFLAG	
MD3799	SV5015 *erfK*::3xFLAG *ycfS*::3xFLAG *ycbB*::3xFLAG *ynhG*::3xFLAG-Km	This study
MD3901	SV5015 *mpl*::3xFLAG *nlpC*::3xFLAG *spr*::3xFLAG-Km	This study
MD4384	SV5015 *slt*::3xFLAG *ldcA*::3xFLAG *amiC*::3xFLAG Δ*scwA*-Km	This study
MD4385	SV5015 *emtA*::3xFLAG *mltD*::3xFLAG *mltC*::3xFLAG Δ*scwA*-Km	This study
MD4386	SV5015 *nagZ*::3xFLAG *mtgA*::3xFLAG Δ*scwA*-Km	This study
MD4389	SV5015 *mrcA*::3xFLAG *dacB*::3xFLAG *dacA*::3xFLAG *STM1910::*3xFLAG Δ*scwA*-Km	This study
MD4390	SV5015 *mrcB*::3xFLAG *dacC*::3xFLAG *STM1836*::3xFLAG *pbpG*::3xFLAG Δ*scwA*-Km	This study
MD4391	SV5015 *mepA*::3xFLAG *dacD*::3xFLAG *mltF*::3xFLAG Δ*scwA*-Km	This study
MD5218	SV5015 *mpl*::3xFLAG *nlpC*::3xFLAG *spr::*3xFLAG Δ*scwA*-Km	This study
MD5219	SV5015 *ecgA*::3xFLAG Δ*scwA*-Km	This study
MD5221	SV5015 *ydhO*::3xFLAG	This study
MD5223	SV5015 *ydhO*::3xFLAG Δ*scwA*-Km	This study
MD5224	SV5015 *ampH::*3xFLAG	This study

MD5225	SV5015 *ynhG*::3xFLAG	This study
MD5226	SV5015 *mltA*::3xFLAG	This study
MD5227	SV5015 *mltB*::3xFLAG	This study
MD5228	SV5015 *ybiS*::3xFLAG	This study
MD5229	SV5015 *ampH*::3xFLAG Δ*scwA*-Km	This study
MD5230	SV5015 *ynhG*::3xFLAG Δ*scwA*-Km	This study
MD5233	SV5015 *ybiS*::3xFLAG Δ*scwA*-Km	This study
MD5242	SV5015 *ΔscwA mltA*::3xFLAG	This study
MD5243	SV5015 *ΔscwA mltB*::3xFLAG	This study
MD4387	SV5015 *erfK*::3xFLAG *ycfS*::3xFLAG *ycbB*::3xFLAG Δ*scwA*-Km	This study
MD5212	SV5015 *slt*::3xFLAG *ldcA*::3xFLAG *amiC*::3xFLAG *mltA*::3xFLAG pUHE21::*scwA*	This study
MD5213	SV5015 *mepA*::3xFLAG *dacD*::3xFLAG *mltF*::3xFLAG *mltB*::3xFLAG pUHE21::*scwA*	This study
MD5214	SV5015 *emtA*::3xFLAG *mltD*::3xFLAG *mltC*::3xFLAG *ybiS*::3xFLAG pUHE21::*scwA*	This study
MD5202	SV5015 *mrcA*::3xFLAG *dacB*::3xFLAG *dacA*::3xFLAG *STM1910*::3xFLAG pUHE21::*scwA*	This study
MD5217	SV5015 *ydhO*::3xFLAG-Km pUHE21::*scwA*	This study
MD5216	SV5015 *mpl*::3xFLAG *nlpC*::3xFLAG *spr*::3xFLAG pUHE21::*scwA*	This study
MD5240	SV5015 *ampH*::3xFLAG pUHE21::*scwA*	This study
MD2553	SV5015 Δ*mltD*-Km	This study
MD5256	SV5015 Δ*mltD* Δ*scwA*-Km	This study
MD2554	SV5015 Δ*ampH*-Km	This study
MD5286	SV5015 Δ*scwA* Δ*ampH*-Km	This study
MD2556	SV5015 Δ*slt*-Km	This study
MD5288	SV5015 Δ*scwA* Δ*slt*-Km	This study
MD2523	SV5015 Δ*dacB*-Km	This study
MD5289	SV5015 Δ*scwA* Δ*dacB*-Km	This study
MD3367	SV5015 Δ*nlpC*-Km	This study
MD5290	SV5015 Δ*scwA* Δ*nlpC*-Km	This study
MD2530	SV5015 Δ*amiC:* -Km	This study
MD5287	SV5015 Δ*scwA* Δ*amiC*-Km	This study
MD5808	SV5015 *emtA*::3xFLAG *mltD*::3xFLAG *mltC*::3xFLAG Δ*scwA*-Km pUHE21::*scwA*	This study
MD5809	SV5015 *emtA*::3xFLAG *mltD*::3xFLAG *mltC*::3xFLAG Δ*scwA*-Km pUHE21::*scwA* Cys30-Ser	This study
MD5810	SV5015 *emtA*::3xFLAG *mltD*::3xFLAG *mltC*::3xFLAG Δ*scwA*-Km pUHE21::*scwA* Cys100-Ser	This study
MD5811	SV5015 *emtA*::3xFLAG *mltD*::3xFLAG *mltC*::3xFLAG Δ*scwA*-Km pUHE21::*scwA* Cys30-Ser Cys100-Ser	This study
**Plasmids**		
pKD13	Kan^R, Amp^R	(Datsenko and Wanner, 2000)
pKD46	γ, β, exo. Amp^R	(Datsenko and Wanner, 2000)
pSUB11	3xFLAG sequence, Kan^R	(Uzzau et al., 2001)
pCP20	FLP^+, Amp^R, Cm^R	(Cherepanov and Wackernagel, 1995)
pKO3Blue	pKO3::*lacZ*, Cm^R	(Solano et al., 2009)
pUHE21-2$lacI^q$	Amp^R	(Soncini et al., 1995)

**Table S2. Oligonucleotides used in this study**	
**Primer**	**Sequence 5' - 3'**
ybaP Fw (2)	ACTGACCCGTTTTTCCAGTG
ybaP Rv (2)	TTGGCCTGCTATCGATCTCT
scwA Fw	GTCACCCAGACTCGCTGCTC
scwA Rv	GTAATCACGCAGAACCCTCAAGCA
copA Fw	CCACCGGGATACCAATGCTG
copA Rv	GTTACTGGCGGTACGCGATCCA
rnpB Fw	TCATCTAGGCCAGCAATCG
rnpB Rv	GGTGAAAGGGTGCGGTAAGA
KO scwA Fw	TGGAGAAAAGGGCGCAATGCGCCCTGACGGGACGTCAACGATCGGTCGGGGTGTAGGCTGGAGCTGCTTC
KO scwA Rv	AAAAAACAACATTTTATAATGTTTTTTTGAATCATAAGGATGTGTTATTTATTCCGGGGATCCGTCGACC
KO dsbA Fw	TCGGAGAGAGTTGATCATGAAAAAGATTTGGCTGGCGCTGGTGTAGGCTGGAGCTGCTTC
KO dsbA Rv	CCGGCGTTCTTTTTATTTTTTATCAACCAAATATTTCACAATTCCGGGGATCCGTCGACC
KO-MltD Fw	TAAAAGGCGCCGGGGGAAGCGGCGCCTTTTGGCTTTTTCGTCGGACAATAGTGTAGGCTGGAGCTGCTTC
KO-MltD Rv	TAACTTCGCCGTTATGATCGGTCGTCTTTTAAGCAACTATTGACACACACATTCCGGGGATCCGTCGACC
KO AmpH Fw	TAACAAAAATGTTGCGCCTCGTCTGATATAGCGCGAGGCGCGACGACTCAGTGTAGGCTGGAGCTGCTTC
KO AmpH Rv	GGTAGTATAAATACGCATAACCACCGTTATTTCACTTATGGACATCACCGATTCCGGGGATCCGTCGACC
KO-slt Fw	ATTCATTACGCGGCATGATGCTGCATTGGATAACAATTGAGGAAGTGCTTGTGTAGGCTGGAGCTGCTTC
KO-slt Rv	GGCCTCTTTCTGCCCCATAAAGTGGCGATAGTAAGCGTCATAGGCCAGAAATTCCGGGGATCCGTCGACC
KO PBP4 Fw	GCGTTGCGCCGTAGTATGACGGCTTGTTTCCAGGGTGTTAGCGCGAGATTGTGTAGGCTGGAGCTGCTTC
KO PBP4 Rv	TAGGCCAGATAAGACGCGTTAGCGCCGCCATCCGGCAATAAATGCACTCAATTCCGGGGATCCGTCGACC
KO-NlpC Fw	AATTTCGACGCTAAATTAATACCAAAATAAAAACAGAGGATTGTTGCGGCGTGTAGGCTGGAGCTGCTTC

KO-NlpC Rv	AACCGGCGATATGTTAAAAATAGACTATAAAATTTATATCGTCTGCGAGGATTCCGGGGATCCGTCGACC
KO-amiC Fw	TGCGGGCAGCATCCTACTTACCCGCGCAATAAACTCGCCGTCATCTCAGGGTGTAGGCTGGAGCTGCTTC
KO-amiC Rv	ATTTGGATGAACTTTGTATGATCTCTATTTAGTTTTTGCTCGGGAGAAGCATTCCGGGGATCCGTCGACC
ForBamHScwAYbaP	CGCTGGATCCATGTTTAGAAAAGCAGTTACGCTTC
RevHinDIIIScwA	CCCTAAGCTTTTATTCGCTGCCGTCGAGATAGGCC
ScwACys30-Ser Rv	GACTGCATTATCGTTGCCaGaTTTTAACTCTGGAGTGGA
ScwACys30-Ser Fw	TCCACTCCAGAGTTAAAAtCtGGCAACGATAATGCAGTC
ScwACys100-Ser Rv	GCATGGCGACGGTAGCCGAgGaAGTTCGTAGGTTGGCATT
ScwACys100-Ser Fw	AATGCCAACCTACGAACTtCcTCGGCTACCGTCGCCATGC
PKD13-1F	AGCACGAGGAAGCGGTCAGCCC
PKD13-2R	CGAGGCAGCGCGGCTATCGTGG
pUHE21 Fw	ATTTATCAAAAAGAGTGTTG
pUHE21 Rv	ACGATGCCATTGGGATATAT
FLAG-ScwAFw	GCCAGCTTGAAATGACCATCGCCAGAACGGCCTATCTCGACGGCAGCGAAGACTACAAAGACCATGACGG
FLAG-ScwARv	TGGAGAAAAGGGCGCAATGCGCCCTGACGGGACGTCAACGATCGGTCGGGCATATGAATATCCTCCTTAG
FLAG-MrcA Fw	ACCATTATTGATAATGGTGAAACACACGAACTGTTCGACTACAAAGACCATGACGG
FLAG-MrcA Rv	GCTAAACACAATAAAAAAGGCGCCGGAGCGCCTTTTTTGACATATGAATATCCTCCTTAG
FLAG-MrcB Fw	GTTGCCGGCTGGATTAAGGAGATGTTCGGCGGCAATGACTACAAAGACCATGACGG
FLAG-MrcB Rv	GACCGGGTAAGCACAGCGCCACCCGGCACTATTACCGTGACATATGAATATCCTCCTTAG
FLAG-PBP 2*-Fw	TGATCCACAGGCTGATACCACACAGCCGGATCAGGCGCCAGACTACAAAGACCATGACGG
FLAG-PBP 2*-Rv	TCCGGCCGTATCCTTGTCTGATGGCGCTTTGCTTATTTGACATATGAATATCCTCCTTAG
FLAG-PBP 3*-Fw	TCTGGTGATGCATGGCAGCCACGTTGCGGTTCCGGGTTCGGACTACAAAGACCATGACGG
FLAG-PBP 3*-Rv	GGGCGCAAGTGTAACGCGAATTGCGCCCCGGGAAAATCCTCATATGAATATCCTCCTTAG
FLAG-MtgA Fw	AGATGCGCCAACTGGGTGGGGAATCTTTTATGACGCGCAACCAGCTTAATGACTACAAAGACCATGACGG

FLAG-MtgA Rv	CGACACAGATCGCAGGCCGGATAAGGCGTTCGCGCCGCCATTCGACAAAACATATGAATATCCTCCTTAG
FLAG-ErfK Fw	AGCGCCGTTCCGGAATGCCGGTCAACATTAGCGCGGGCAGGCCTGGCCTTGACTACAAAGACCATGACGG
FLAG-ErfK Rv	TACTAATACAGATACTCCAGCCCATGAAAACCAGCAATGCGACAGGATATCATATGAATATCCTCCTTAG
FLAG-YbiS Fw	TCGTACAACAGGCAGTTCAGGATCGTTCCGGGATGCCGGTTCGTCTGAACGACTACAAAGACCATGACGG
FLAG-YbiS Rv	GCCGTAAGAGAAGCCCGATAGTATCTATCGGGCTTCGCAGGCAGAGTCTGCATATGAATATCCTCCTTAG
FLAG-YcfS Fw	GAATGCCAGTGGATGTAACCCGTCAAGCAGAGGCGAAACCGCAGTCGCTGGACTACAAAGACCATGACGG
FLAG-YcfS Rv	AAAGCCTCGCGATGCATCGCGAGGCTTTTTTGTCGGTCAGCTATTCACGCCATATGAATATCCTCCTTAG
FLAG-YcbB Fw	CAGCGCGATCCAGCGCACAAATTGTGCCAAAGGTTGAACAATTAATCAGGGACTACAAAGACCATGACGG
FLAG-YcbB Rv	CCCCGTTGAAGCCAAACATTAGGACAACATATTTACCAGAACTTCTTCATCATATGAATATCCTCCTTAG
FLAG-YnhG Fw	CCGCTCAGAACGGTTTTGTCGGGGAAGAGGGGCAAACGCGCGCGACGCAGGACTACAAAGACCATGACGG
FLAG-YnhG Rv	AAATGGCGCACATCGTGCGCCATTTTTTTGTCCGTCGTTTGCTGCAAAGGCATATGAATATCCTCCTTAG
FLAG-PBP 4 Fw	CGCTGGTACGCTTCGAGAGTCGGTTGTATAAGGATATTTATCAGAATAACGACTACAAAGACCATGACGG
FLAG-PBP 4 Rv	TTGTAGGCCAGATAAGACGCGTTAGCGCCGCCATCCGGCAATAAATGCACCATATGAATATCCTCCTTAG
FLAG-PBP 5 Fw	TTGTAGGCCAGATAAGACGCGTTAGCGCCGCCATCCGGCAATAAATGCACCATATGAATATCCTCCTTAG
FLAG-PBP 5 Fw	TCGGTAAAATCATTGATTACATTAAATTAATGTTCCATCACTGGTTTGGAGACTACAAAGACCATGACGG
FLAG-PBP 5 Rv	CTGATGCATAGTATATGGGGACGAAAATCACACTTTCAAGCGTTCAATTTCATATGAATATCCTCCTTAG
FLAG-PBP 6 Fw	GCTGATGAAACTCCATCAGTGGTTTGGCAGTTGGTTCTCGGACTACAAAGACCATGACGG
FLAG-PBP 6 Rv	CCGTAGCCGGATGCGACGCGCACCCGGCTACGGAGTTATTCATATGAATATCCTCCTTAG
FLAG-PBP 6b Fw	TATGTTCTCTCGTCTGAGCGACTATTTTCAGCATAAAGCGGACTACAAAGACCATGACGG
FLAG-PBP 6b Rv	GACACCTCAGATGACGGTGAACGGTGTGTGTGACAACGGCCATATGAATATCCTCCTTAG
FLAG-PBP 7 Fw	AGCCAGCGCCAGTGCCGGAGCGCAAACCGCACAGAACGATGACTACAAAGACCATGACGG
FLAG-PBP 7 Rv	TGAAGCCCGGCGGCGCGATGCCTGCCGGGCCTGCGGCGACCATATGAATATCCTCCTTAG
FLAG-AmpH Fw	ATTTGGTTACTGAGCTAAGCGGCAATAAACCGATTGCTATTCCTGCATCCGACTACAAAGACCATGACGG
FLAG-AmpH Rv	TATTAACAAAAATGTTGCGCCTCGTCTGATATAGCGCGAGGCGCGACGACCATATGAATATCCTCCTTAG

FLAG-MepA Fw (1)	TTACTATTTATCGTCGTCATCTTTGTAGTCGATATCATGATCTTTATAATCACCGTCATGGTCTTTGTAGTCGAGTACATGCTCATCCAGTA
FLAG-MepA Fw	CGCGGATCCGCGATAACAGCAGGCCGCCAACG
FLAG-MepA Rv (1)	CGCGGATCCGCGGTCATGTTTATCCAGCGGTT
FLAG-MepA Rv (2)	GACTACAAAGACCATGACGGTGATTATAAAGATCATGATATCGACTACAAAGATGACGACGATAAATAGTAATGGACAATTTTTATGATCTG
FLAG-YdhO Fw A	ACGTCATTATGTCGGCGCCCGCCGGGTGATGACGCCTAAAACAATTCGCGACTACAAAGACCATGACGG
FLAG-YdhO Rv	GGTGGCGCAAAAGAAGAAGTTATCCTGTCGTTAAACGACAGGATAAAATACATATGAATATCCTCCTTAG
FLAG-Spr Fw	CGTACTGGAAAAAACGCTACAATGAAGCGCGTCGAGTTCTGAGCCGCAGTGACTACAAAGACCATGACGG
FLAG-Spr Rv	TTTTATGTCGTCTCATCAGGTAAGCCAAGGGAGGTGCTGCCTGATGAAGACATATGAATATCCTCCTTAG
FLAG-NlpC Fw	CGCTGGATAATGTGTACTGGCGAAAAAACTTCTGGCAGGCACGGCGAATTGACTACAAAGACCATGACGG
FLAG-NlpC Rv	AACCGGCGATATGTTAAAAATAGACTATAAAATTTATATCGTCTGCGAGGCATATGAATATCCTCCTTAG
FLAG-STM1940 Fw	AGCTGATATCACTGGCTGGTAAGGTTCTGTTTAAGCTTGGTTATATAGAAGACTACAAAGACCATGACGG
FLAG-STM1940 Rv	CGCGCACGGTTGCCTTAATCATGTCTTCCGCGATAACACAGTTTGTCAACCATATGAATATCCTCCTTAG
FLAG-MltA Fw	GCGTCTGGGTGTTAAAAAGCGCGCCGGGCGCCGGTAACGTGTTTAGCGGCGACTACAAAGACCATGACGG
FLAG-MltA Rv	CCGATAGCGATCGCGTTATCGGGCAATCCGGTTGTCGCAGAATACCACAACATATGAATATCCTCCTTAG
FLAG-MltB Fw	CGATGGCGGTTTGGCAACTGGGCCAGGCGGTAGCGCTGGCGCGGGTGCGCGACTACAAAGACCATGACGG
FLAG-MltB Rv	CAAAAAGCCATAATAATAGCGGAGGGGGTGCTCCCCTCCCTAAGCGTAAACATATGAATATCCTCCTTAG
FLAG-MltC Fw	GCCGCTATCTGTATAAGGTGAATTCCGCCCAGCGATCGTATCGCCGCCGAGACTACAAAGACCATGACGG
FLAG-MltC Rv	TTACCTTTCCCGCCTCTGTCATTTGCCAACCAGAGGCGGGATGCGATGGTCATATGAATATCCTCCTTAG
FLAG-MltD Fw	CGGGCGATCAGCTTACGTTGTTTGTGAAGGACAATAGCACGCCAGACTCCGACTACAAAGACCATGACGG
FLAG-MltD Rv	TAAAAGGCGCCGGGGGAAGCGGCGCCTTTTGGCTTTTTCGTCGGACAATACATATGAATATCCTCCTTAG
FLAG-EmtA Fw	CGCAGGCGCCGCGCTATATCTGGAAGCTTCAGCAGGCGCTGGACGCCATGGACTACAAAGACCATGACGG
FLAG-EmtA Rv	CGAGGCCCGAGAAAGAGCGAATAAAGTGCGAGAATAATCTTCACGCTAAACATATGAATATCCTCCTTAG
FLAG-MltF Fw	TGACGCATTCGCCGTCTTTACTGTTTACGCCGCAGAAGAAAGAAGAAAAAGACTACAAAGACCATGACGG

FLAG-MltF Rv	CGCAATGCCGGATAAGACGTTGCGCGTCGCCATCCGATAAGACCATCGCGCATATGAATATCCTCCTTAG
FLAG-Slt Fw	TGGGGCAGAAAGAGGCCTTAATGAGCGACTCTGAGTGGCAGCGACGGTATGACTACAAAGACCATGACGG
FLAG-Slt Rv	CGCCGTTTGTACTGTTACACGAGTACAAATGATAACATAACCCGTCTGCACATATGAATATCCTCCTTAG
FLAG-AmiC Fw	CGGGCATTAAAGCGTATTTTGCCGATGGGGCGACGCTGGCGAGAAGAAGTGACTACAAAGACCATGACGG
FLAG-AmiC Rv	TGCGGGCAGCATCCTACTTACCCGCGCAATAAACTCGCCGTCATCTCAGGCATATGAATATCCTCCTTAG
FLAG-NagZ Fw	CCCAGCTCAACCAGTTACATGAACGCTGGCAGGAAGAAAAAGCAGGTCATGACTACAAAGACCATGACGG
FLAG-NagZ Rv	TCAAAACCGTGTAAATAAATAATCATCTGGCACCCTCACGAAAACTCGAGCATATGAATATCCTCCTTAG
FLAG-LdcA Fw	CGCGCCAGGGGACTCAACTCACTTTATCTGGCCATCCTACGCTGCAATTGGACTACAAAGACCATGACGG
FLAG-LdcA Rv	ACATAATAACGGCCAGGGCAGCGTTTTACTTAGCCTGTCGGGGACTTTTTCATATGAATATCCTCCTTAG
FLAG-Mpl Fw	AGAAATTGCTGGATGGGCTGGCGAAAAAAGCTCAGAACGTAACAGCGTATGACTACAAAGACCATGACGG
FLAG-Mpl Rv	ATGCCCCATATAAAACAGCCAGCCACAAGGCTGGCTGACCGCATAGTCATCATATGAATATCCTCCTTAG
FL ybaP Fw	TACCGCACATCCTGTAGAAAA
FL scwA Rv	TCTGAAGATCCGAAGCGAAAG
FL dsbAFw	TACAATTAACGCCAATGTATTAATCGGAGAGAGAGTTGATC
FL dsbARv	AACATCTTATAAAAACGCCGGTCAGTGACCGGCGTTCTTT
FL-1A-Fw	AGCCATAACGGTCGATCTCC
FL-1A-Rv	AGGGCCGAATTGCCTGATGG
FL-1B-Fw	TCGTGACGTGTTATACGTTGCCTC
FL-1B-Rv	GACGTAAGCGTCTTATTCGGCCTA
FL-2* Fw	AGGCCTGACTCCGGATAACGGA
FL-2* Rv	ACCTCTATGCCTTACGGGCAG
FL-3* Fw	TGGCGCTGTGGACATATAACG
FL-3* Rv	GCCTCAGATATAAAGCCTCGCT
FL-YcbB Fw	CAGCGCAGTCATGCGGAGTA

FL-YcbB Rv	ACGAAAGGCACCTTAACTGCCGA
FL-PBP 4 Fw	GGAGTTCCCTATGGGTCATCG
FL-PBP 4 Rv	GCTTACGCTTATCCGGCCTAC
FL-PBP 5 Fw	TGGTTTTCATGTCAGCTCCG
FL-PBP 5 Rv	AAGTCAGATGCCTGCCAGTTATG
FL-6 Fw	TCTTTAACGTCGTGAGTGCGT
FL-6 Rv	CAGTAAATTCGGCAAGGTACG
FL-6b Fw	TACGGTCACAAGGACAGCGTGA
FL-6b Rv	TCCGTAGCATGGTTGACGGCT
FL-7 Fw	CGACTTAATCGTTCTGTGCGGT
FL-7 Rv	TGATCCTGTGGGGATACGGCT
FL-AmpH Fw	TGATCAGTATTAACCGTTTGCCC
FL-AmpH Rv	TCCATCACGAAAACCCGC
FL-YdhO Fw	GTGCGCGTAAGGATAGGGTA
FL-YdhO Rv	CGCGTTCGTGTTTAGCATTA
FL-STM1940 Fw	TGTCTTCCGCGATAACACAG
FL-STM1940 Rv	GTGATCCGCATCATGCTTTA
FL-MltA Rv	TGCGGACCAGACGACAGATA
FL-MltB Fw	TTTGAACCGTATGACGGCG
FL-MltB Rv	GAAGAATACCTGTCACCATACCGG
FL-NupG Fw	GAACGATAAGATTCAGGCCGC
FL-NupG Rv	AACCCACAGGATGCCAACAG
FL-MltD Fw	GAGTTGCAGGATGACGGTTC
FL-MltD Rv	CATTCGCTAACTTCGCCGTT

FL-EmtA Fw	TCCATCAGCGAGCGTAAGGT
FL-EmtA Rv	GTCCGTACCGGCAAAACG
FL-Slt Fw	GCATGATGCTGCATTGGATA
FL-Slt Rv	AGCCGATGAATAAGGGGAAT
FL-AmiC Fw	GCGGAGTATAGTGCGCATCC
FL-AmiC Rv	CTGTTTTTGCCCTCTGTGCA
YrbL Fw	GATTACCGATTTCAACGGCG
YhbL Rv	ATCCTGCGCCAGCATATAGG
YeeO Fw	GCTAAAGGCGACCACCACCGTA
CobT Rv	ATGACCGGCGTGATGCTTGG
YbiR Fw	TTGGCGCACTGTCTCACCTG
YbiT Rv	GCTCATTTCCGGCAAGGCGTA
YcfR Fw	CGCCGTTGAAGTTCAGGCAACG
Mfd Rv	AAACGCCGTTGATGCGCTGAA
YnhA Fw	CCAACGGCATTATCGAATTGCA
LppB Rv	GTCTTCCGATGTACAGACGCTGAG
AroC Fw	CGAACCGGTATTTGACCGAC
YfcA Rv	TGAAAATCACCAGGATGGGC
YeiU Fw	CCGCCTTTATGCTGCGTTAT
Rtn Rv	GTGAATACGCCGCTGTCATT
BtuD Fw	CCTTGCTGCCGTTGTACTAC
YdiV Rv	AAGTCACTAATTGCAGGCGG
STM2566 Fw	CCGACGAAACCGTTTGCGTA
YfhC Rv	AGATCACGAATACTGGATGCGCCA

ThiK Fw	GCGCCTTTGCATATGGATGT
YcfP Rv	GCATATTCTTCCGGGCGAT
YcgO Rv	GCGTCAGTGGAACCGACTATC
Fbp Fw	CGTCGATGTTAGAAGAGCCATCC
STM4417 Rv	GGCTTTTCCGGGGTAGGTTCC

References

Cherepanov, P. P., and Wackernagel, W. (1995). Gene disruption in *Escherichia coli*: TcR and KmR cassettes with the option of Flp-catalyzed excision of the antibiotic-resistance determinant. *Gene* 158, 9–14. doi:10.1016/0378-1119(95)00193-a.

Datsenko, K. A., and Wanner, B. L. (2000). One-step inactivation of chromosomal genes in *Escherichia coli* K-12 using PCR products. *Proc Natl Acad Sci U S A* 97, 6640–5. doi:10.1073/pnas.120163297.

Dominguez-Bernal, G., Pucciarelli, M. G., Ramos-Morales, F., Garcia-Quintanilla, M., Cano, D. A., Casadesus, J., et al. (2004). Repression of the RcsC-YojN-RcsB phosphorelay by the IgaA protein is a requisite for *Salmonella* virulence. *Mol Microbiol* 53, 1437–49. doi:10.1111/j.1365-2958.2004.04213.x.

Hanahan, D. (1983). Studies on transformation of *Escherichia coli* with plasmids. *J Mol Biol* 166, 557–580. doi:10.1016/s0022-2836(83)80284-8.

Rico-Pérez, G., Pezza, A., Pucciarelli, M. G., de Pedro, M. A., Soncini, F. C., and García-del Portillo, F. (2016). A novel peptidoglycan D,L-endopeptidase induced by *Salmonella* inside eukaryotic cells contributes to virulence. *Mol Microbiol* 99, 546–556. doi:10.1111/mmi.13248.

Solano, C., García, B., Latasa, C., Toledo-Arana, A., Zorraquino, V., Valle, J., et al. (2009). Genetic reductionist approach for dissecting individual roles of GGDEF proteins within the c-di-GMP signaling network in *Salmonella. Proc Natl Acad Sci U S A* 106, 7997–8002. doi:10.1073/pnas.0812573106.

Soncini, F. C., Véscovi, E. G., and Groisman, E. A. (1995). Transcriptional autoregulation of the *Salmonella typhimurium* phoPQ operon. *J Bacteriol* 177, 4364–4371. doi:10.1128/jb.177.15.4364-4371.1995.

Uzzau, S., Figueroa-Bossi, N., Rubino, S., and Bossi, L. (2001). Epitope tagging of chromosomal genes in *Salmonella*. *Proc Natl Acad Sci U S A* 98, 15264–9. doi:10.1073/pnas.261348198.

Vivero, A., Banos, R. C., Mariscotti, J. F., Oliveros, J. C., Garcia-del Portillo, F., Juarez, A., et al. (2008). Modulation of horizontally acquired genes by the Hha-YdgT proteins in *Salmonella enterica* serovar Typhimurium. *J Bacteriol* 190, 1152–6. doi:10.1128/JB.01206-07.
